# Supplementary material for: Gene Expression-Based Identification of Antigen-Responsive CD8+ T Cells on a Single-Cell Level
Source: Front Immunol. 2019 Nov 6;10:2568. doi: 10.3389/fimmu.2019.02568 (PMC6851025; doi:10.3389/fimmu.2019.02568)
Supplement: Figure S1 — Impaired MHC class I multimer detection of CD8+ T cells responding to their cognate antigen. [file Presentation_1.pdf]

Fuchs YF, Sharma V *et al.*: Gene Expression-Based Identification of Antigen-Responsive CD8+ T cells on a Single-Cell Level.

Supplementary Figures and Tables:

Fig. S1

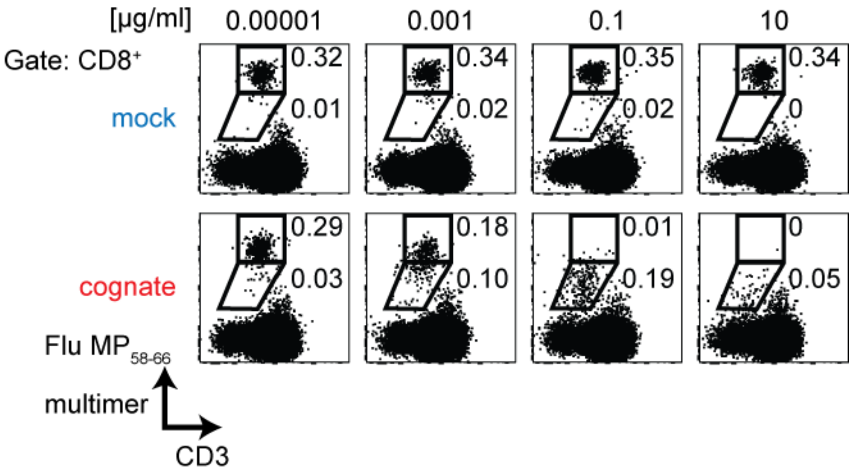

Fig. S1: Impaired MHC class I multimer detection of CD8+ T cells responding to their cognate antigen. Representative flow cytometry dot plots of PBMCs stained with HLA-A2 multimers loaded with Flu MP_{58-66} peptide after incubating PBMCs for 20 h in the presence of mock ($IGRP_{265-273}$) or cognate (Flu MP_{58-66}) peptide at the indicated concentrations. Plots show 1×10^5 cells in the CD8 gate and the frequencies of cells showing strong or intermediate positive staining with the multimer.

Fig. S2

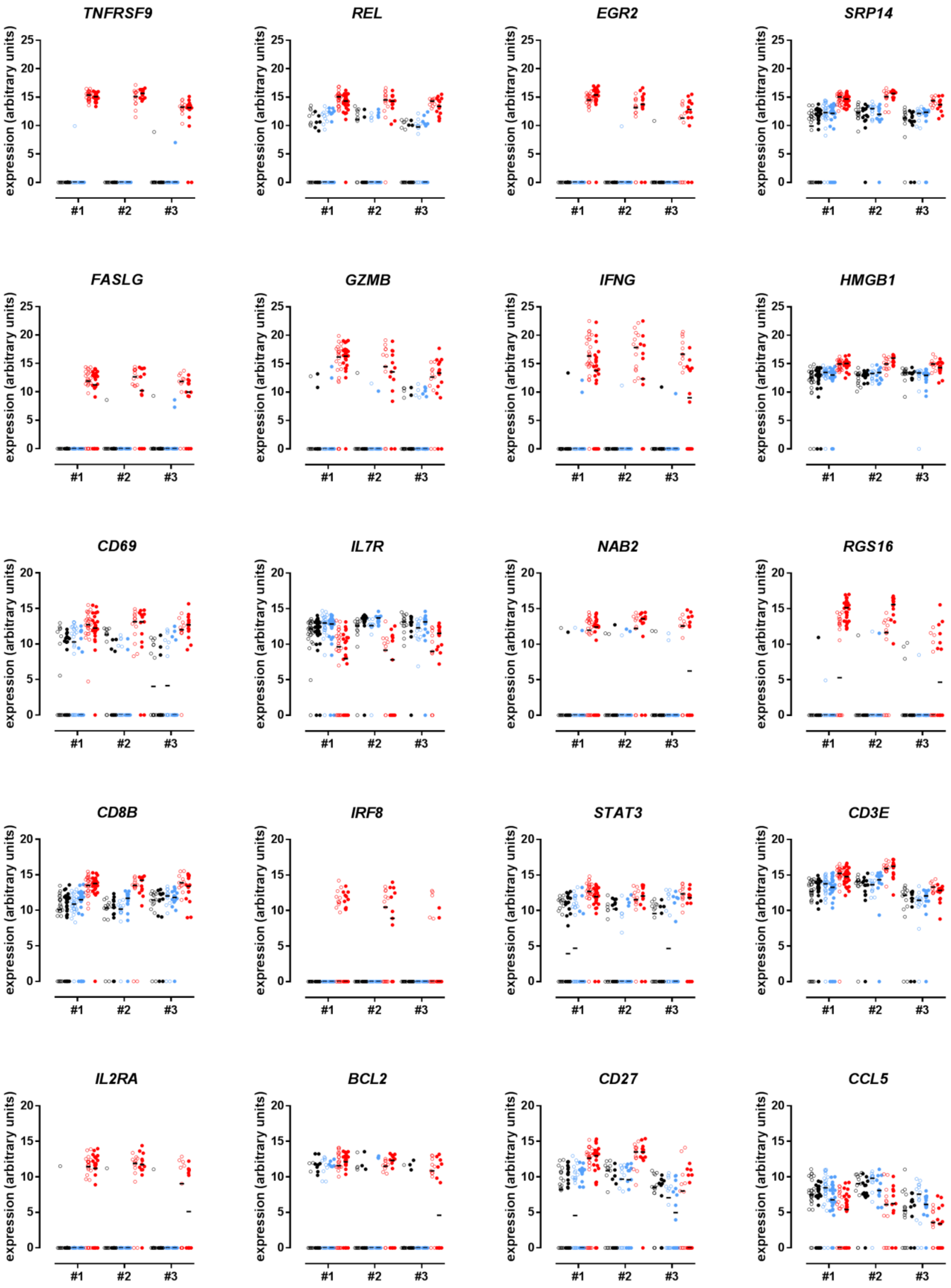
TNFRSF9
REL
EGR2
SRP14
FASLG
GZMB
IFNG
HMGB1
CD69
IL7R
NAB2
RGS16
CD8B
IRF8
STAT3
CD3E
IL2RA
BCL2
CD27
CCL5
expression (arbitrary units)
0
5
10
15
20
25
#1
#2
#3

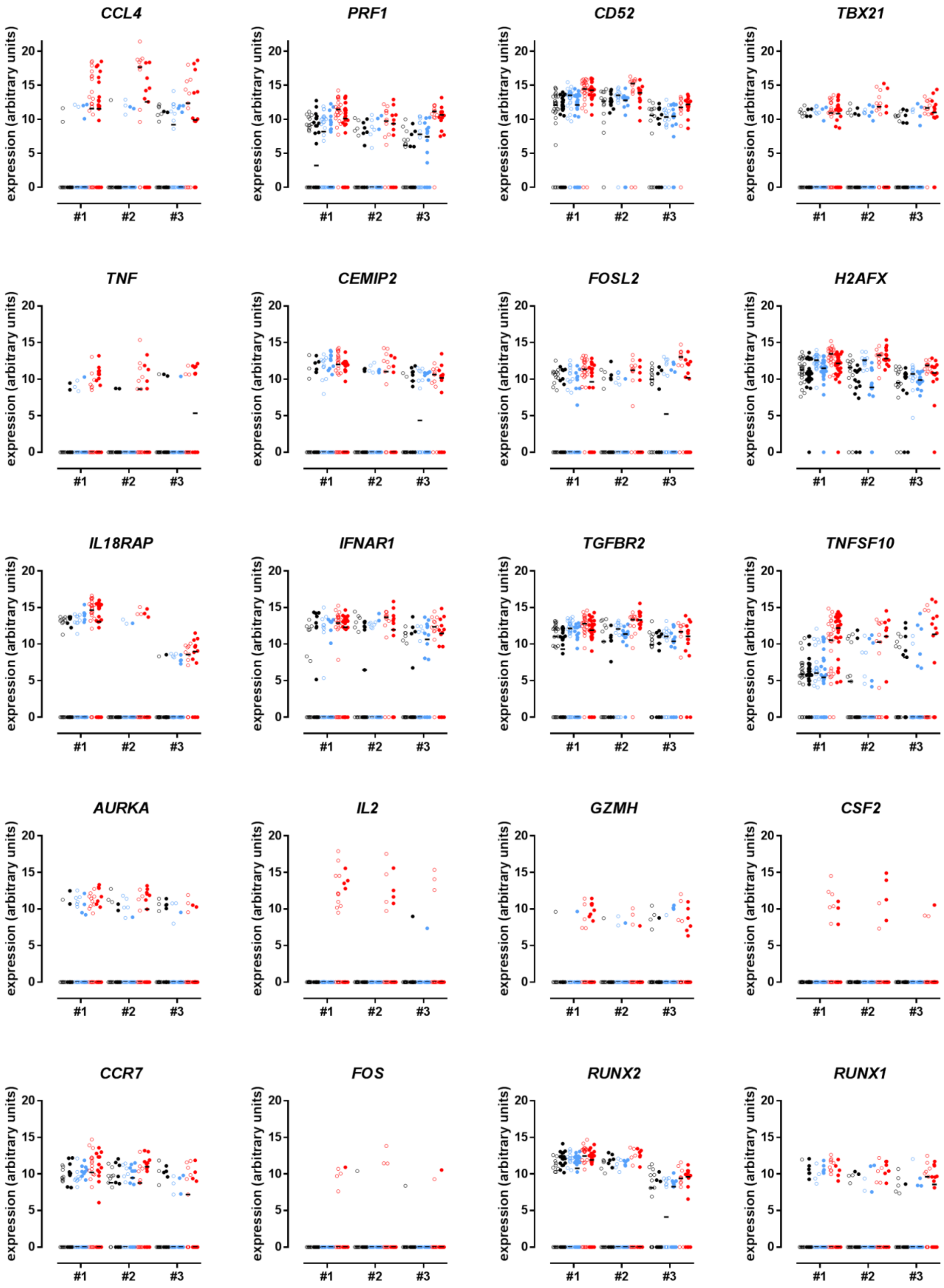
CCL4
PRF1
CD52
TBX21
TNF
CEMIP2
FOSL2
H2AFX
IL18RAP
IFNAR1
TGFBR2
TNFSF10
AURKA
IL2
GZMH
CSF2
CCR7
FOS
RUNX2
RUNX1
expression (arbitrary units)
0
5
10
15
20
#1
#2
#3

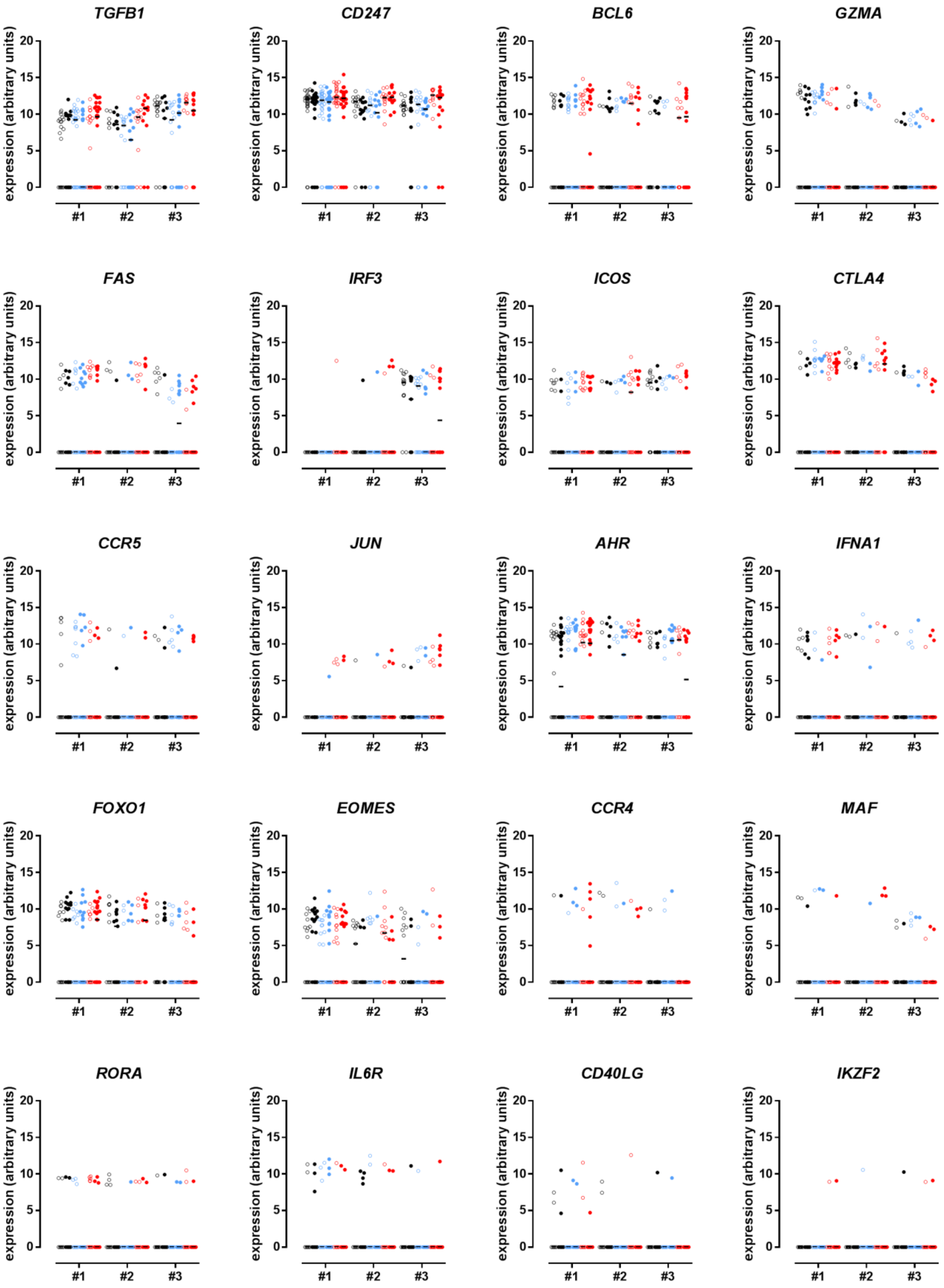
TGFB1
CD247
BCL6
GZMA
FAS
IRF3
ICOS
CTLA4
CCR5
JUN
AHR
IFNA1
FOXO1
EOMES
CCR4
MAF
RORA
IL6R
CD40LG
IKZF2
expression (arbitrary units)
0
5
10
15
20
#1
#2
#3

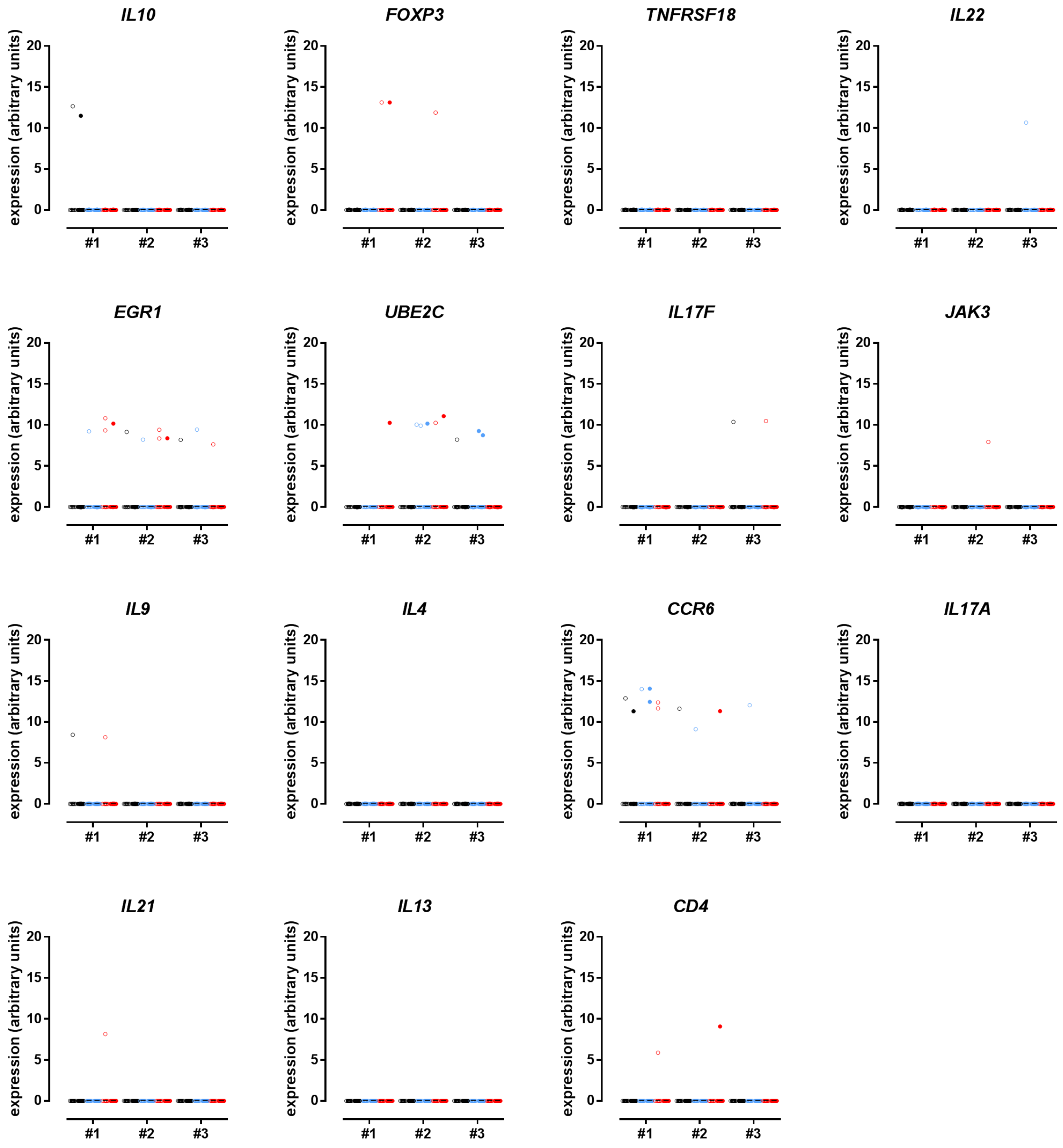

Fig. S2: Gene expression analysis of Flu MP$_{58\text{-}66}$-directed CD8$^+$ T cells after incubation with peptide-loaded K562/A*0201 cells or autologous PBMCs. Scatter plots are shown for 75 genes determined using the qPCR-based targeted gene expression approach. Plots show the expression in individual Flu MP$_{58\text{-}66}$-directed single-cell-sorted CD8$^+$ T cells from donors #1–3 incubated overnight with DMSO (black) or mock peptide (blue) as control stimuli or with cognate peptide (red) in the presence of K562/*0201 cells (open symbols) or autologous PBMCs (filled symbols) for antigen presentation in the dye-based CD8$^+$ T cell activation assay. The qPCR primers are listed in Table S9. The following numbers of cells were analyzed:

	K562/A*0201			PBMC		
	DMSO	mock	cognate	DMSO	mock	cognate
**Donor #1**	26	28	26	25	25	28
**Donor #2**	13	13	13	13	13	13
**Donor #3**	14	14	11	11	14	14

Fig. S3

A K562/A*0201: mock vs. cognate

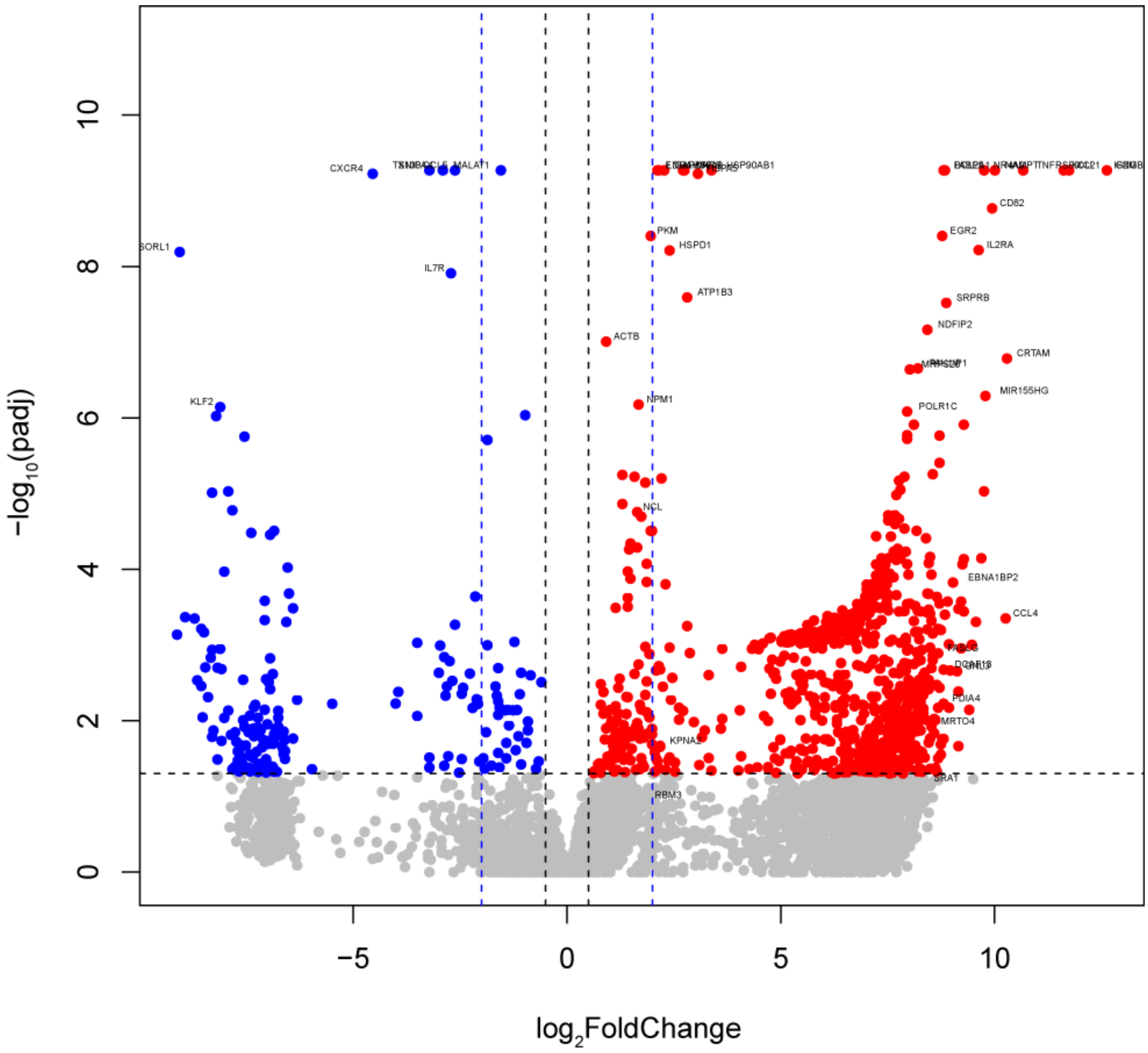

B K562/A*0201: DMSO vs. mock

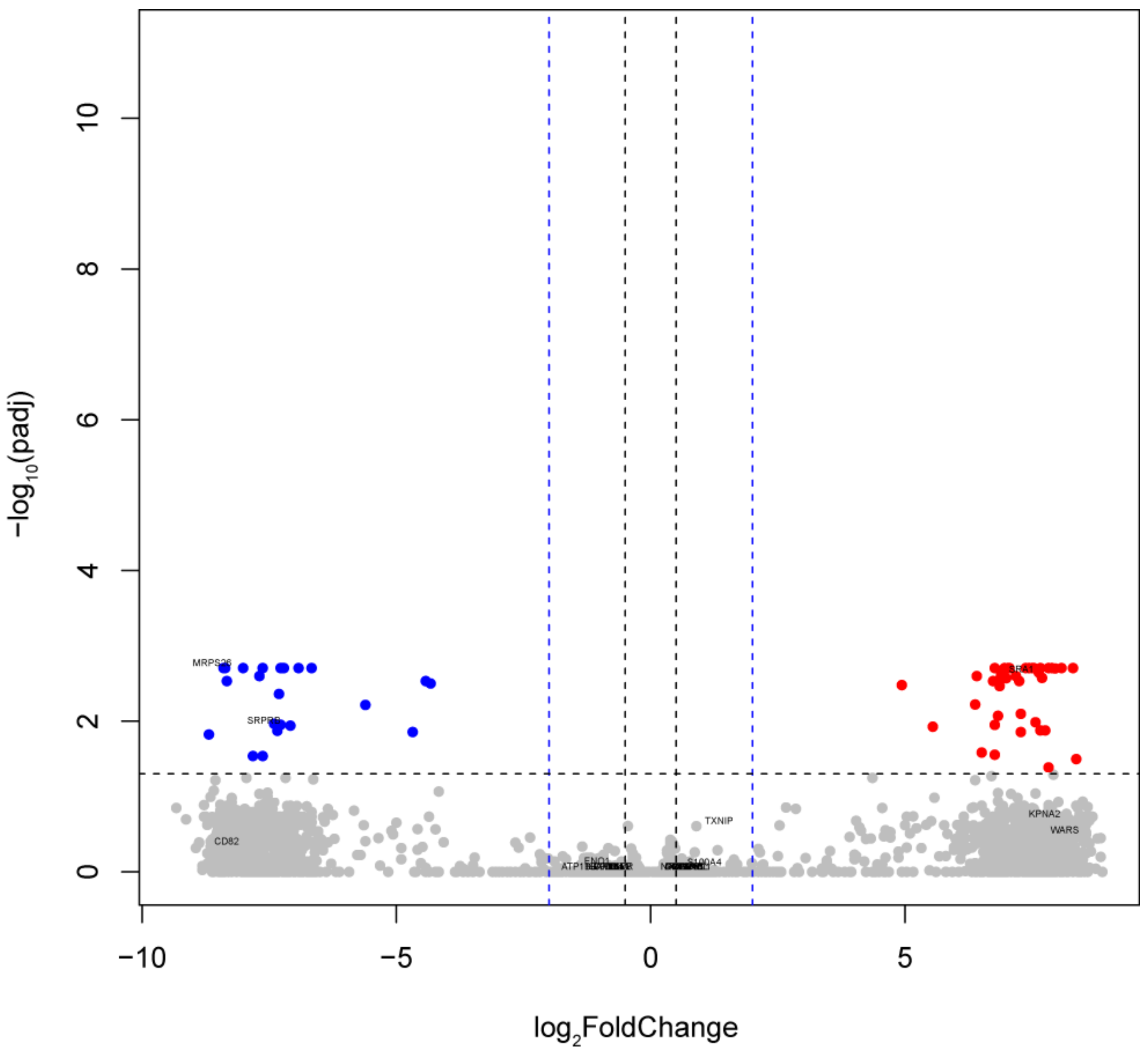

C K562/A*0201: DMSO vs. cognate

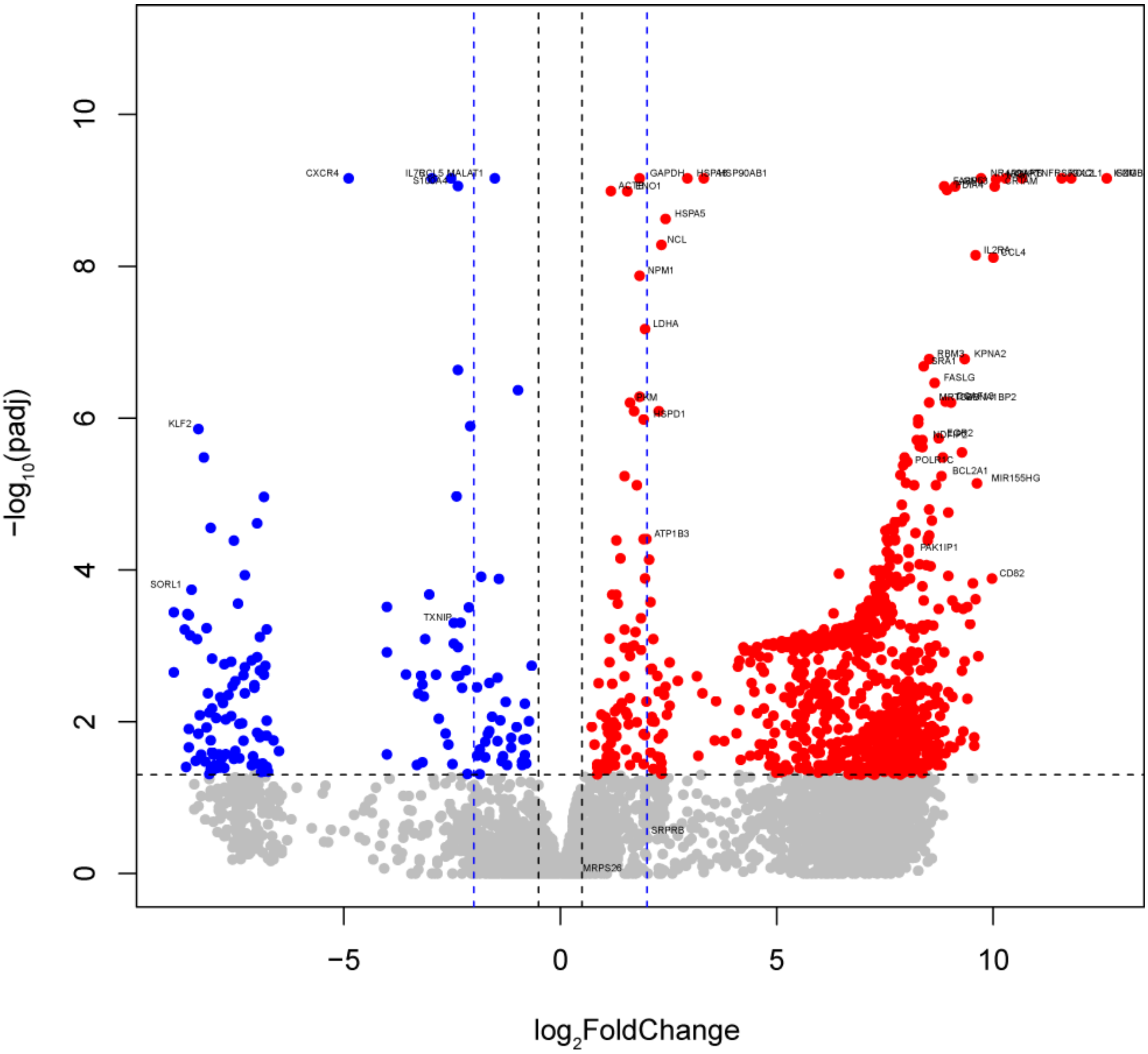

D PBMC: mock vs. cognate

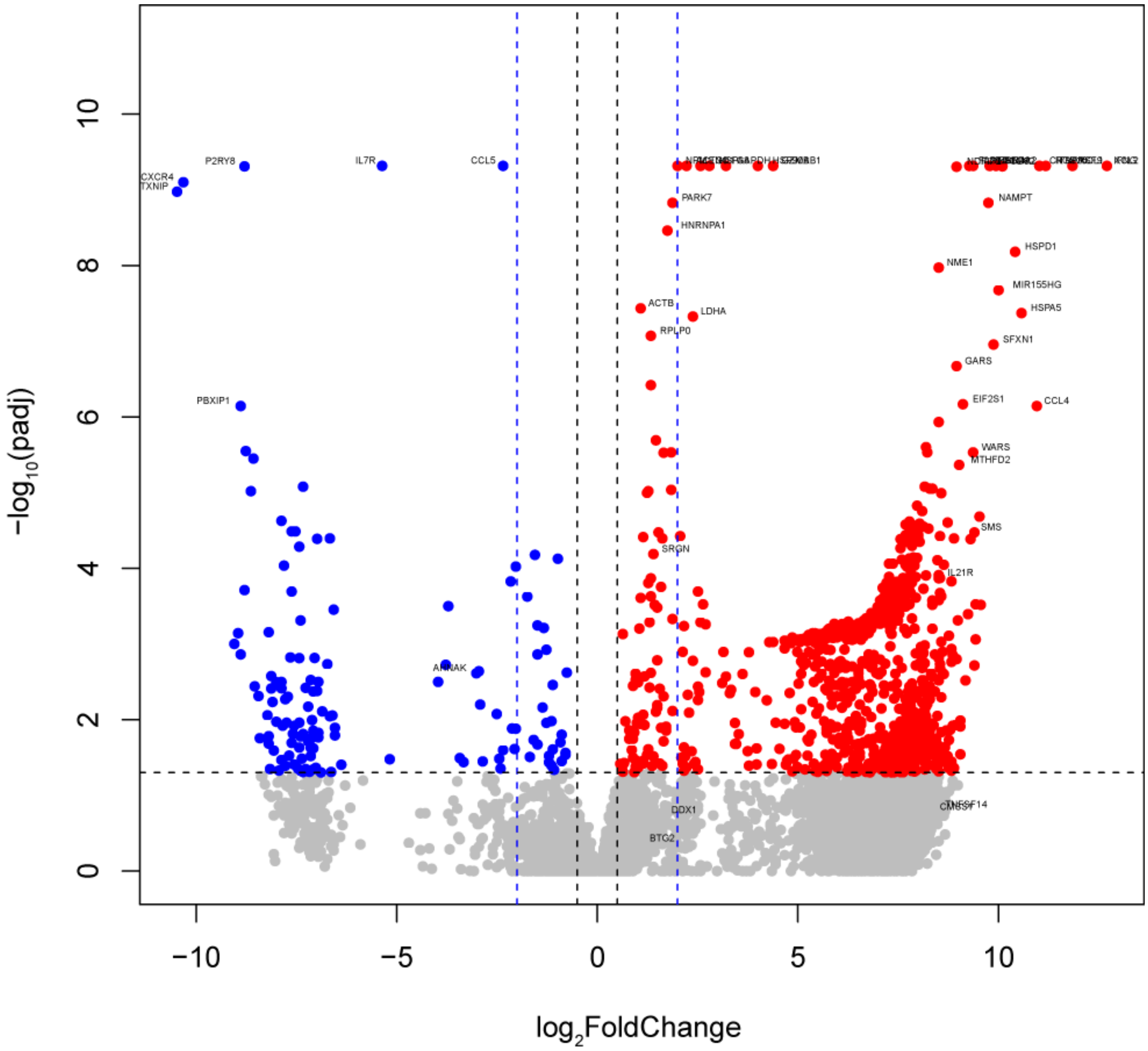

E PBMC: DMSO vs. mock

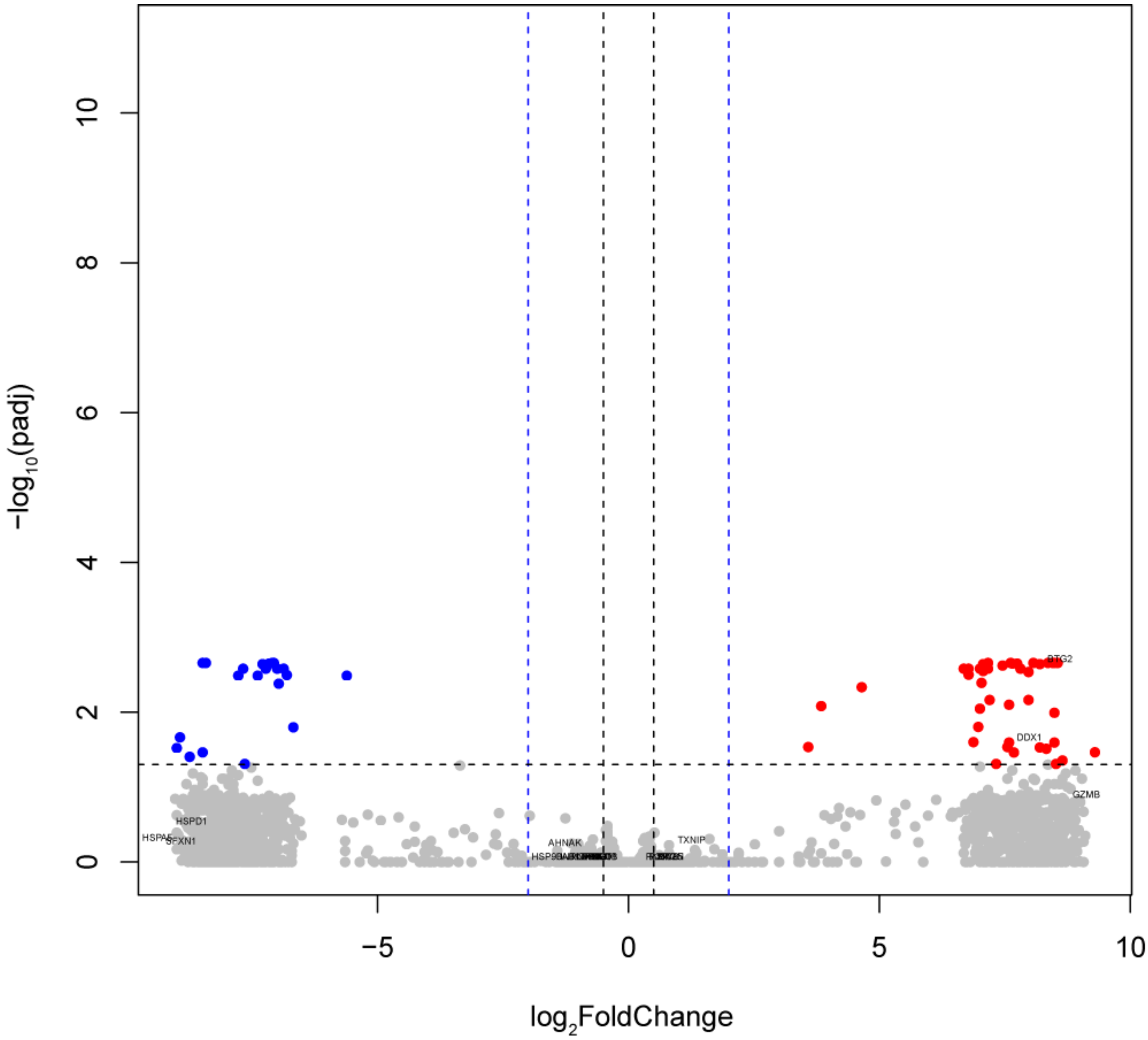

F PBMC: DMSO vs. cognate

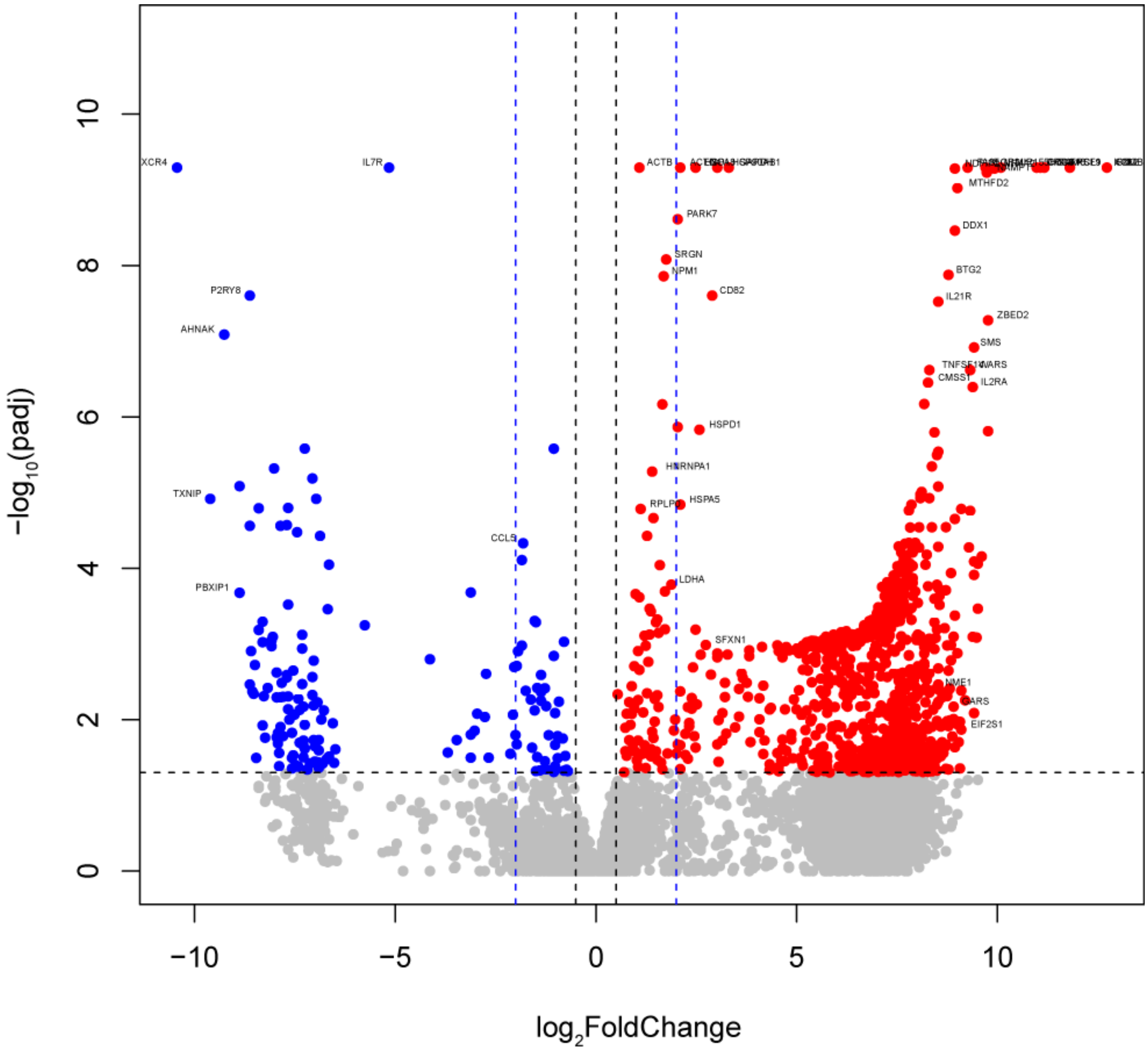

Fig. S3: Comparison of gene expression between cognate peptide-stimulated and control-stimulated Flu MP$_{58\text{-}66}$-directed $CD8^+$ T cells. (**A–F**) Volcano plots of scRNAseq data for Flu MP$_{58\text{-}66}$-directed cells from donor #1. Cells were single-cell-sorted from the dye-based $CD8^+$ T cell activation assay following overnight incubation with peptide solvent (DMSO), mock peptide (IGRP$_{265\text{-}273}$), or cognate peptide (Flu MP$_{58\text{-}66}$) in the presence of K562/A*0201 cells or autologous PBMCs for antigen presentation. The thresholds (fold change = 0.5 [black] and 4 [blue]; adjusted p = 0.05 [black]) are shown as dashed lines. Genes upregulated in cells responding to their cognate peptide are marked in red and downregulated genes in blue. The

gene symbols for the combined top 50 ranked differentially expressed genes in both comparisons (DMSO vs. cognate and mock vs. cognate) using the same cells for antigen presentation are shown. Volcano plots comparing cells incubated with peptide solvent and mock peptide (DMSO vs. mock) are also included as control conditions.

Fig. S4

A Donor #4: mock vs. cognate

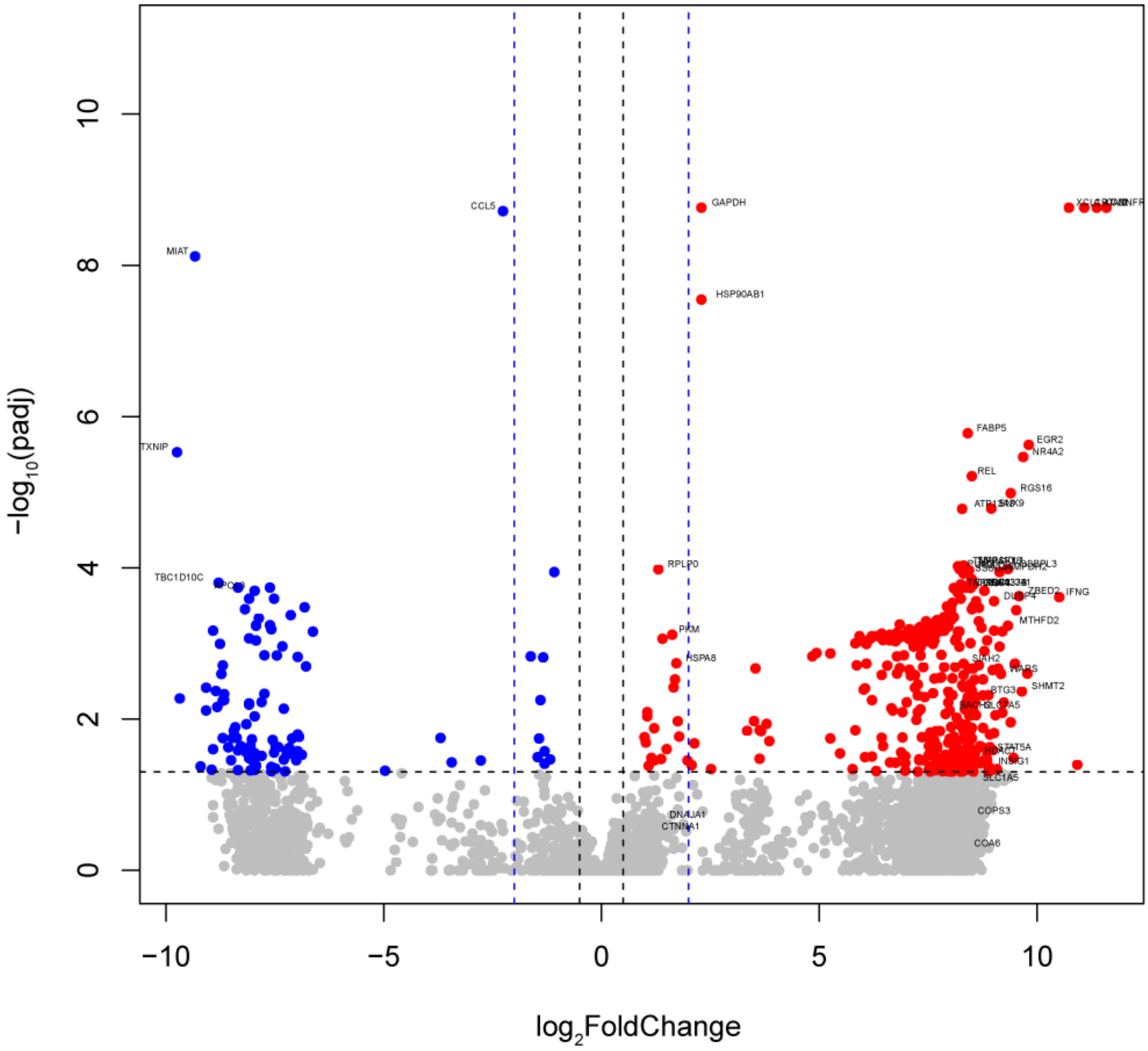

B Donor #4: DMSO vs. mock

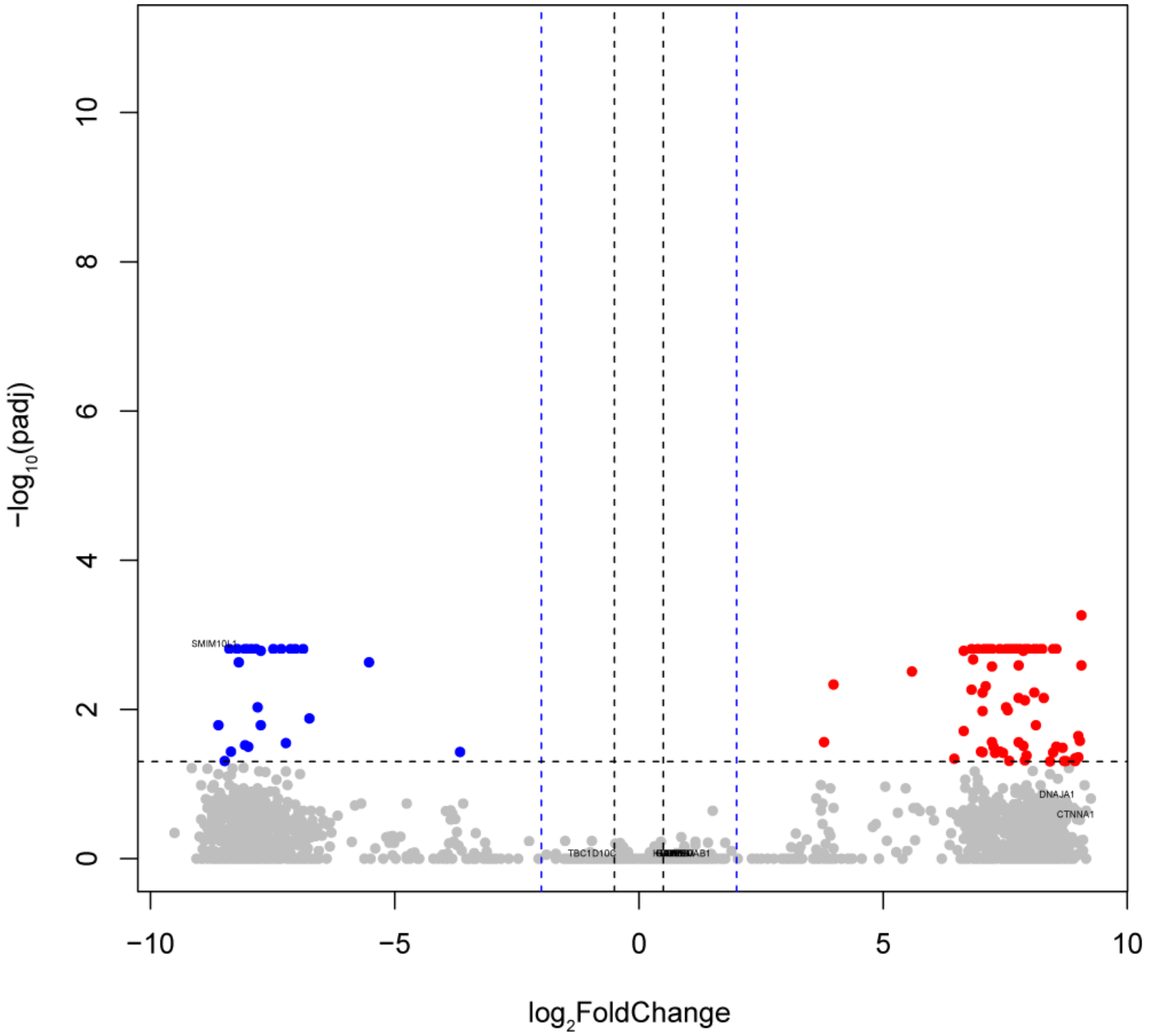

C Donor #4: DMSO vs. cognate

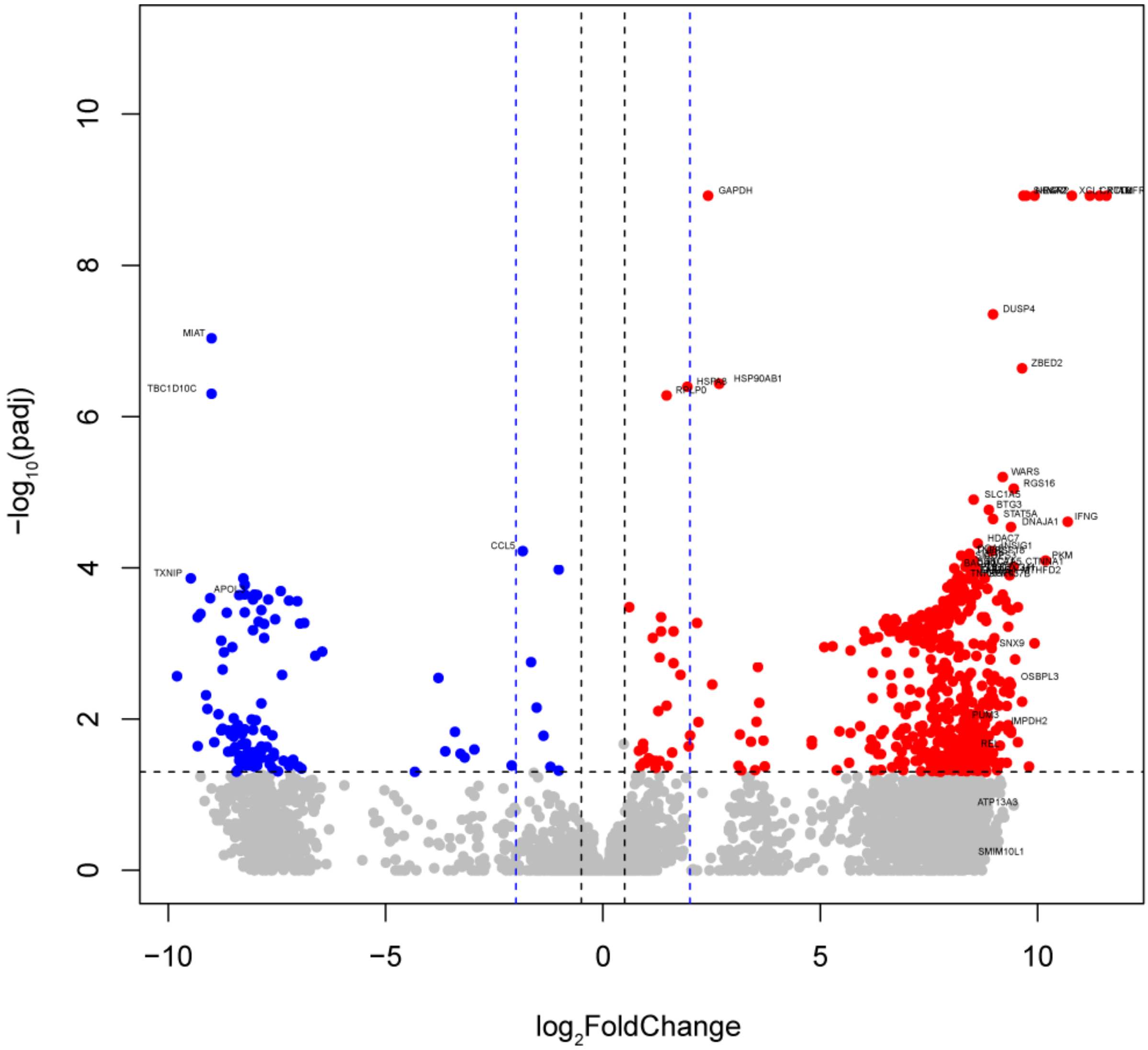

D Donor #5: mock vs. cognate

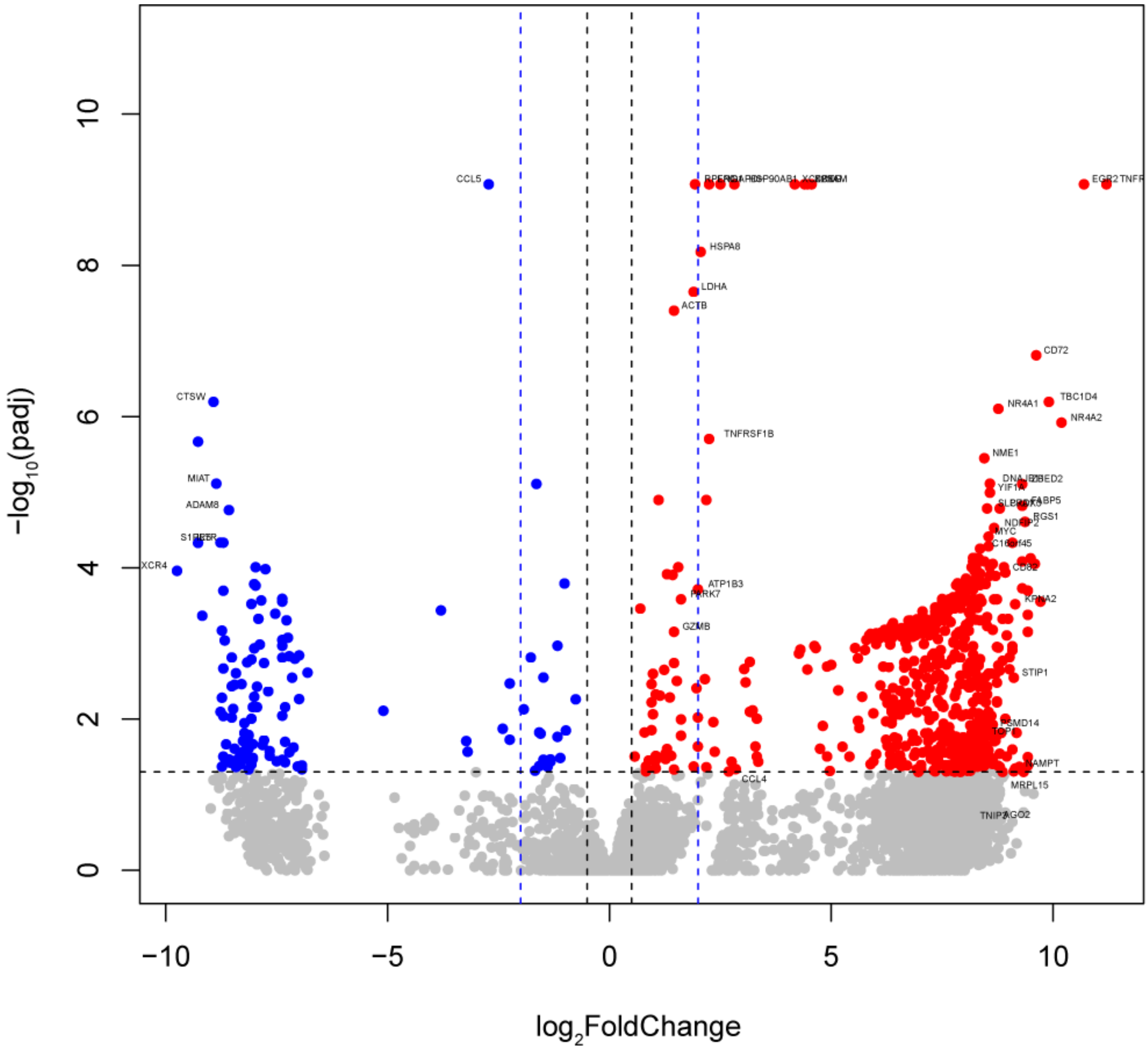

E Donor #5: DMSO vs. mock

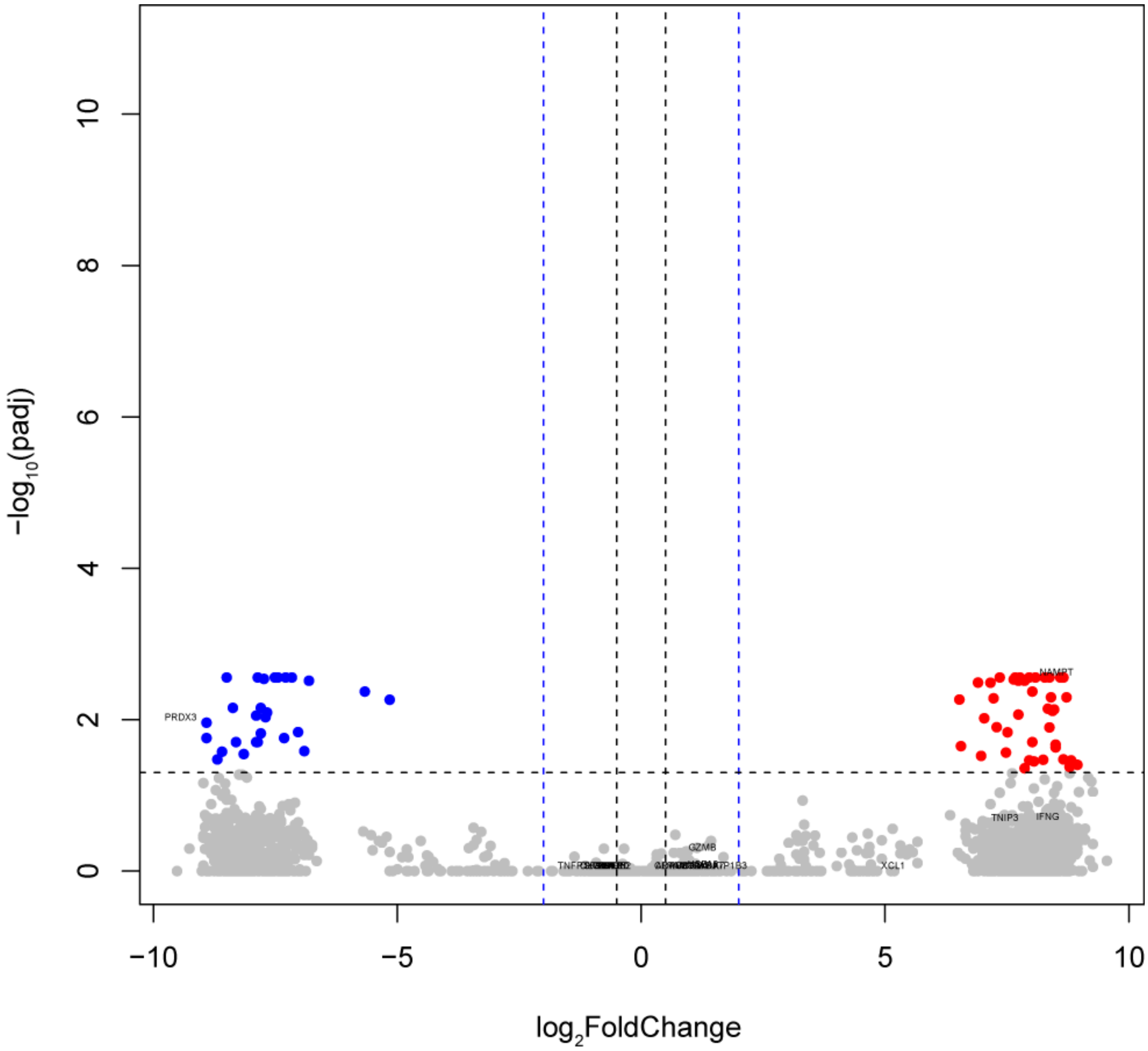

F Donor #5: DMSO vs. cognate

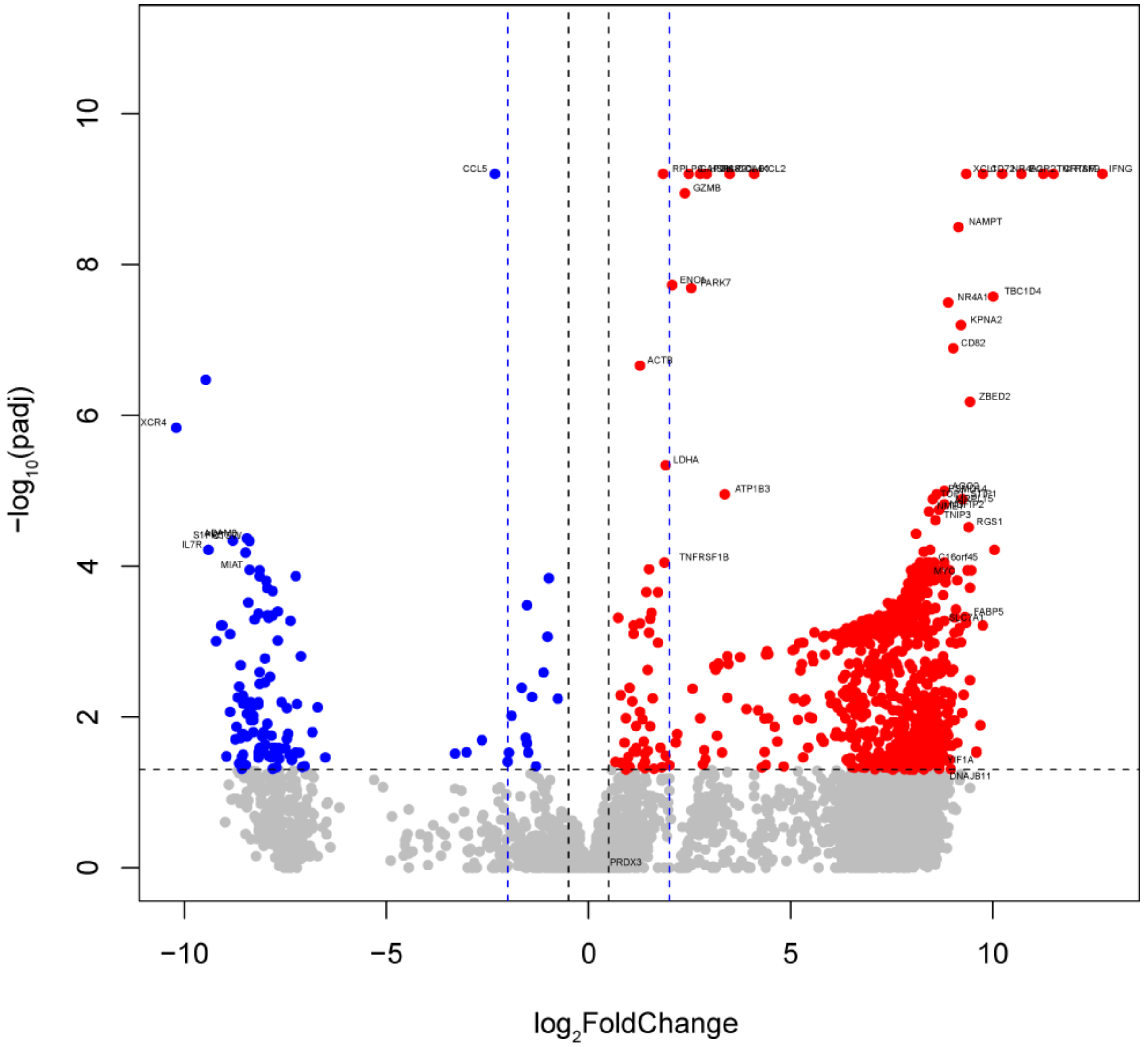

G Donor #6: mock vs. cognate

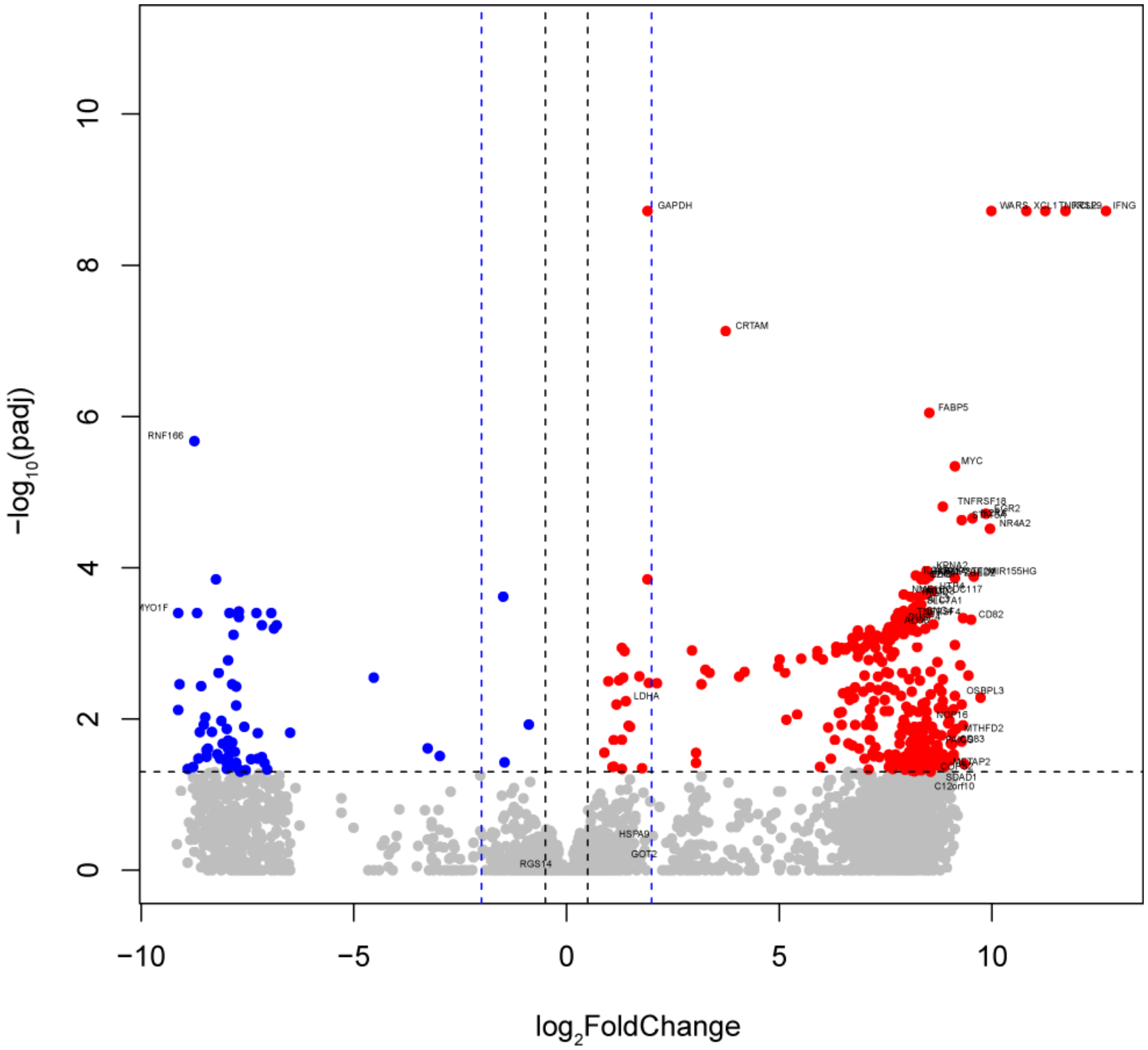

H Donor #6: DMSO vs. mock

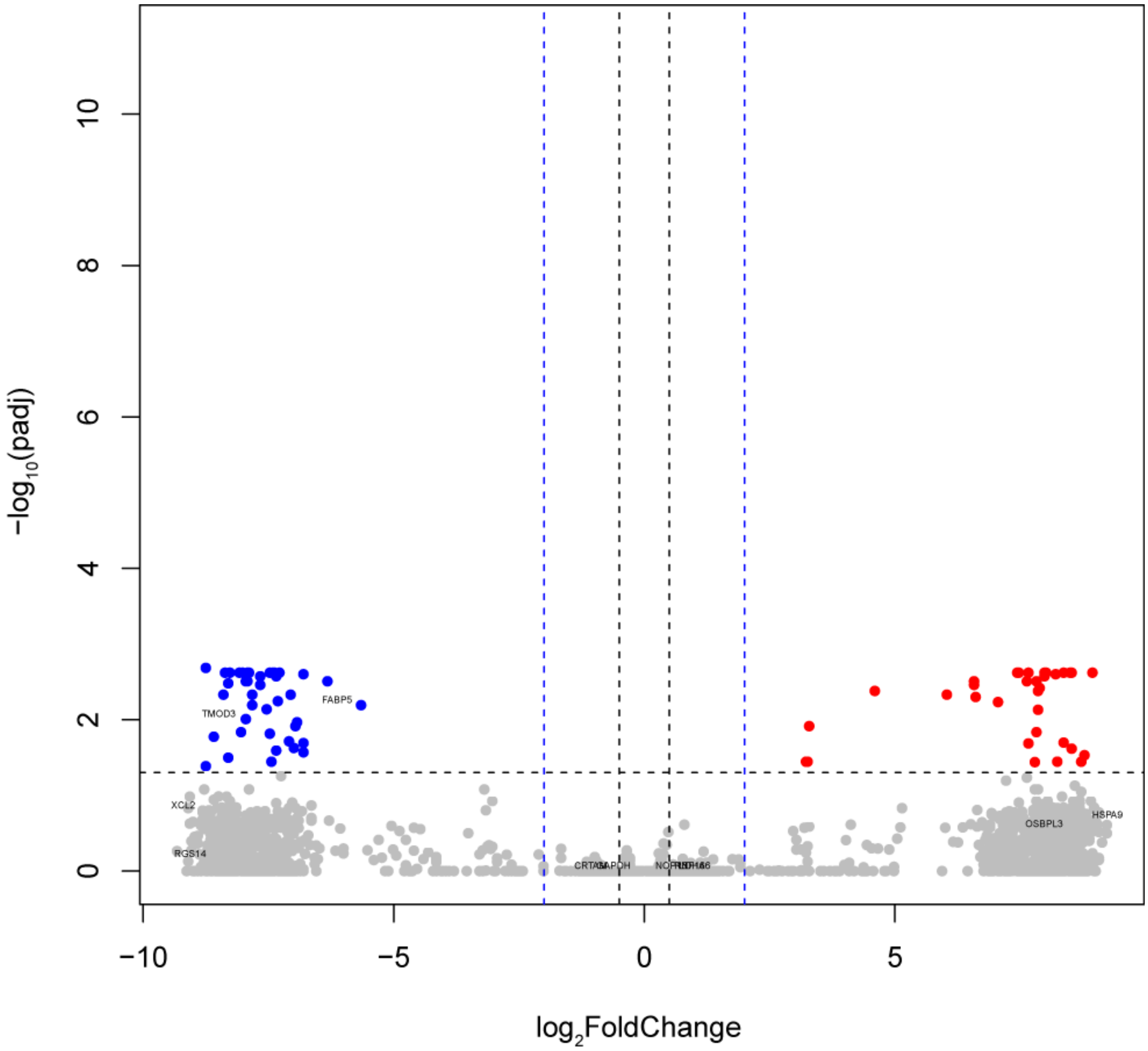

I Donor #6: DMSO vs. cognate

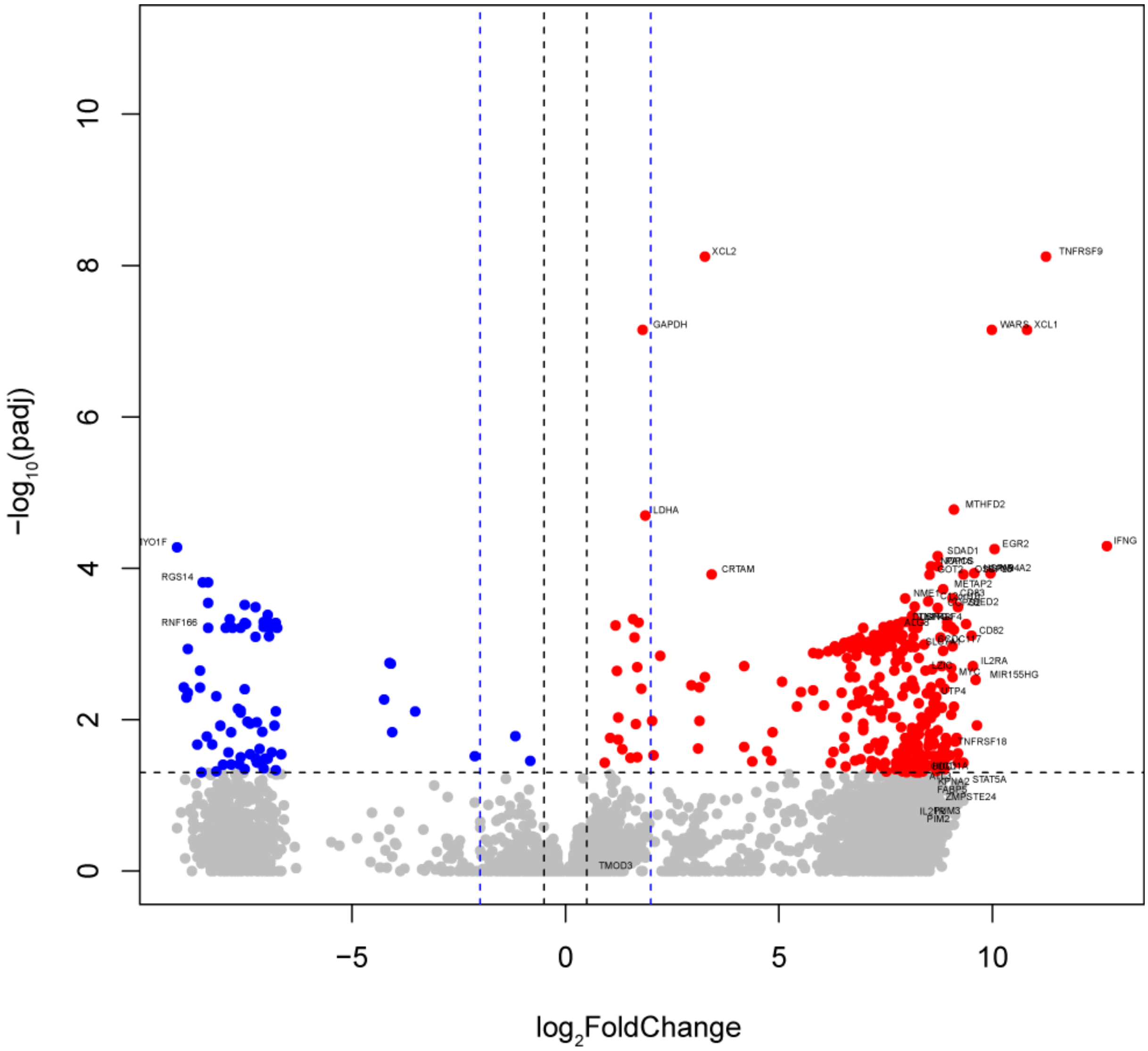

Fig. S4: Comparison of gene expression between cognate peptide-stimulated and control-stimulated CMV pp65$_{495\text{-}503}$-directed CD8$^+$ T cells. (**A–I**) Volcano plots of scRNAseq data of CMV pp65$_{495\text{-}503}$-directed cells from donors #4–6 are shown. Cells were single-cell-sorted from the dye-based CD8$^+$ T cell activation assay following overnight incubation with peptide solvent (DMSO), mock peptide (Flu MP$_{58\text{-}66}$), or cognate peptide (CMV pp65$_{495\text{-}503}$) in the presence of autologous PBMCs for antigen presentation. The thresholds (fold change = 0.5 [black] and 4 [blue]; adjusted p = 0.05 [black]) are shown as dashed lines. Genes upregulated in cells responding to their cognate peptide are marked in red and downregulated genes in blue. The gene symbols for the combined top 50 ranked differentially expressed

genes in both comparisons (DMSO vs. cognate and mock vs. cognate) are shown. Volcano plots comparing cells incubated with peptide solvent and mock peptide (DMSO vs. mock) are included as control conditions. Genes upregulated in cells stimulated with mock peptide are marked in red and downregulated genes in blue.

Fig. S5

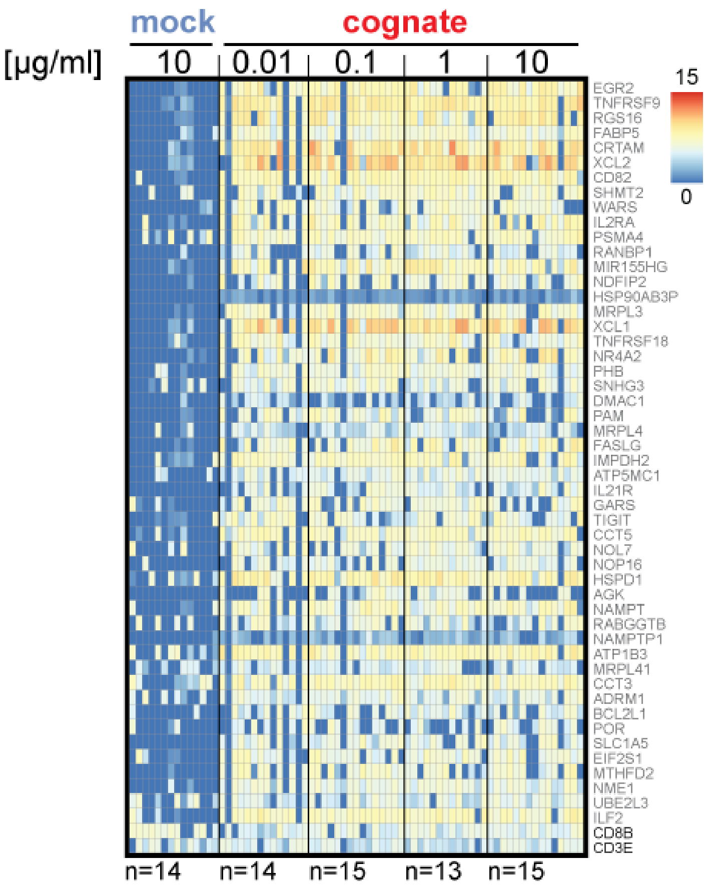

Fig. S5: Discriminative ability of antigen-responsive cells across a wide range of cognate peptide concentrations. Heatmap showing gene expression in single-cell-sorted Flu $MP_{58\text{-}66}$-directed cells incubated overnight with mock ($IGRP_{265\text{-}273}$) or titrated amounts of cognate (Flu $MP_{58\text{-}66}$) peptide in the dye-based $CD8^+$ T cell activation assay using autologous PBMCs for antigen presentation. Results are shown for the top 50 ranked separator genes for cognate peptide-responsive $CD8^+$ T cells of donor #1 and PBMC-based stimulation (see Table S5).

Fig. S6

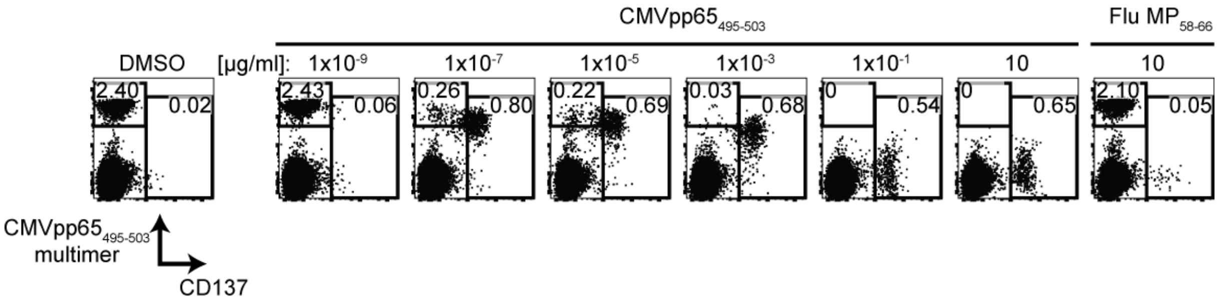

Fig. S6: Impaired MHC class I multimer detection and upregulation of CD137 in $CD8^+$ T cells responding to their cognate antigen. Representative flow cytometry dot plots of PBMCs stained with HLA-A2 multimers loaded with CMV $pp65_{495\text{-}503}$ peptide after the PBMCs were incubated for 20 h in the presence of CMV $pp65_{495\text{-}503}$ (cognate) or Flu $MP_{58\text{-}66}$ peptide (mock) at the indicated concentrations. Plots show 0.5 × 10^4 cells in the CD8 gate and the frequencies of multimer-stained cells (left gate) or CD137-expressing cells (right gate).

Fig. S7

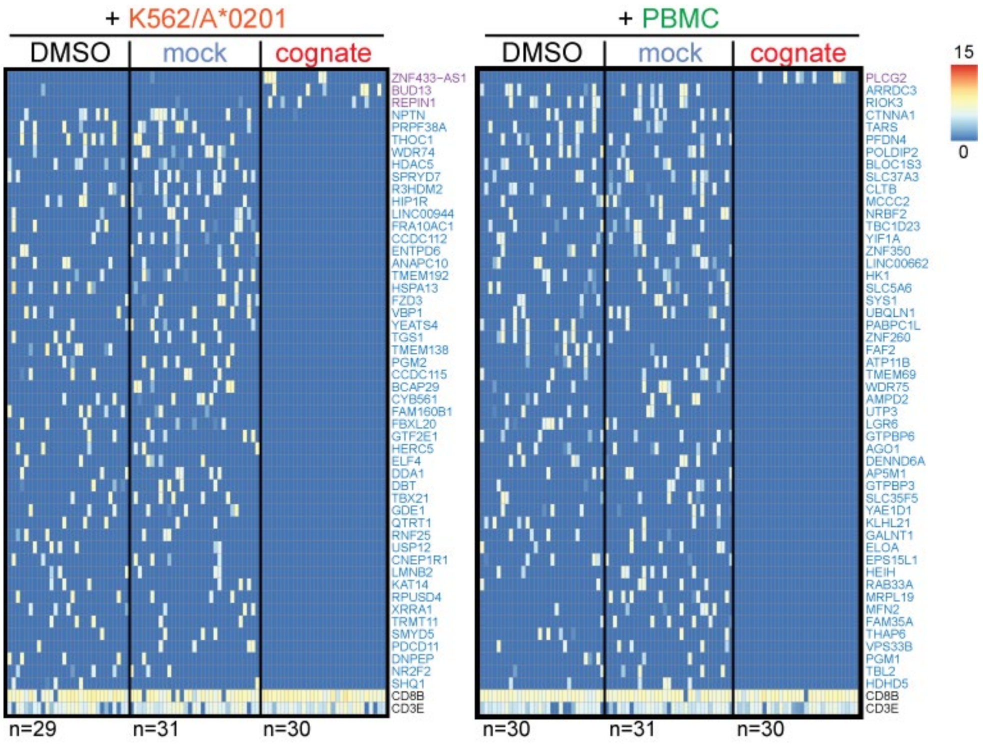

Fig. S7: IGRP$_{265\text{-}273}$-directed CD8^{+} T cells markedly differ from virus-directed cells in their response to cognate antigen. Heatmaps showing the top 50 ranked differentially expressed genes in autoantigen-directed CD8^{+} T cells incubated with cognate peptide (IGRP$_{265\text{-}273}$) relative to control cells incubated with DMSO or mock peptide (Flu MP$_{58\text{-}66}$). Cells were derived from a donor with type 1 diabetes and data were generated by scRNAseq. The top 50 genes were ranked separately for the K562/A*0201 (left) and autologous PBMC (right) antigen presentation conditions.

Fig. S8

A

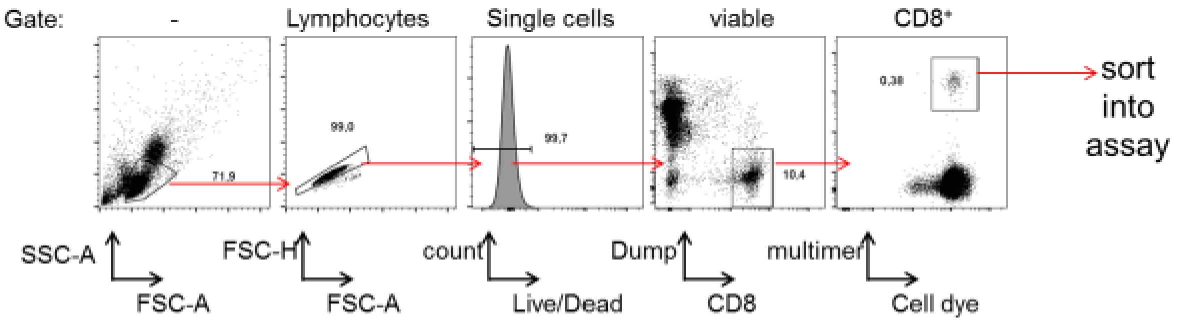
Gate:
-
Lymphocytes
Single cells
viable
CD8⁺
sort into assay
SSC-A
FSC-A
FSC-H
FSC-A
count
Live/Dead
Dump
CD8
multimer
Cell dye

B

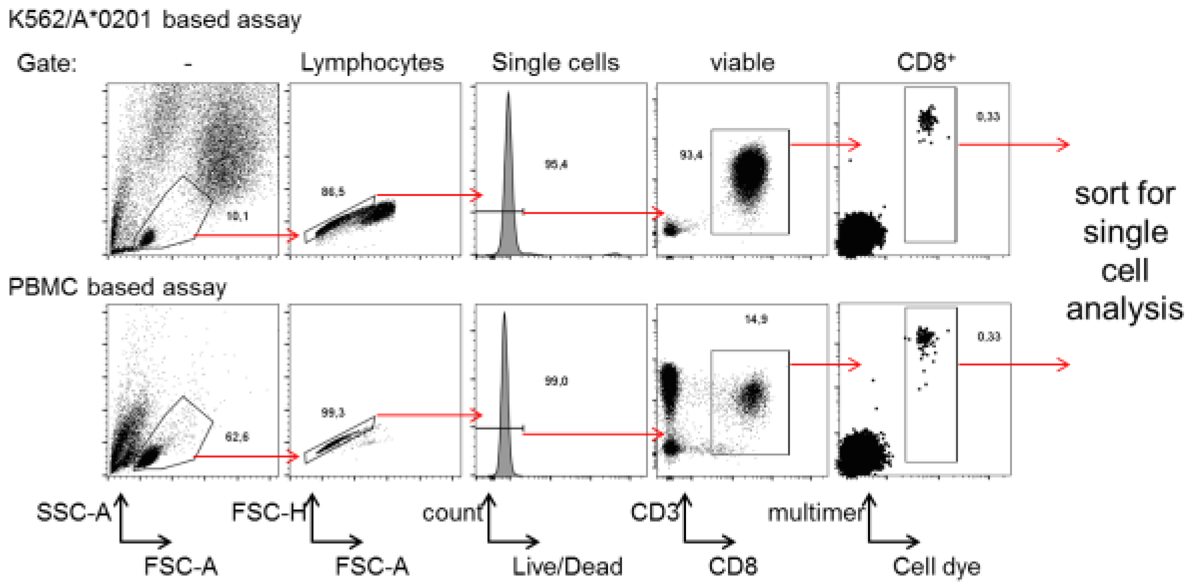
K562/A*0201 based assay
Gate:
-
Lymphocytes
Single cells
viable
CD8⁺
PBMC based assay
sort for single cell analysis
SSC-A
FSC-A
FSC-H
FSC-A
count
Live/Dead
CD3
CD8
multimer
Cell dye

C

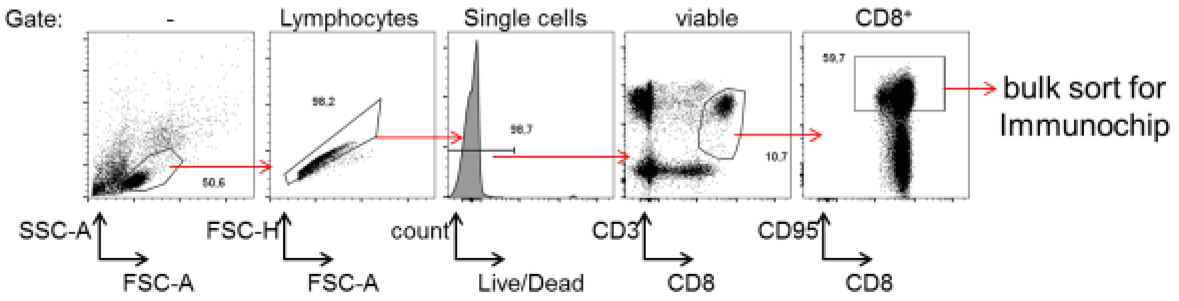
Gate:
-
Lymphocytes
Single cells
viable
CD8⁺
bulk sort for Immunochip
SSC-A
FSC-A
FSC-H
FSC-A
count
Live/Dead
CD3
CD8
CD95
CD8

Fig. S8: Gating strategies for the flow sorting experiments. Representative flow cytometry plots and gates are shown for sorting of (**A**) multimer-positive CD8$^+$ T cells into the dye-based activation assay, (**B**) dye-stained antigen-directed CD8$^+$ T cells from K562/A*0201- or PBMC-based assays for single-cell gene expression analysis, and (**C**) CD95$^+$CD8$^+$ memory T cells for immunochip analysis.
