# Supplementary material for: Gene Expression-Based Identification of Antigen-Responsive CD8+ T Cells on a Single-Cell Level
Source: Front Immunol. 2019 Nov 6;10:2568. doi: 10.3389/fimmu.2019.02568 (PMC6851025; doi:10.3389/fimmu.2019.02568)
Supplement: Table S1 — Overview of statistical results of the targeted gene expression analysis. [file Table_1.pdf]

Table S1

	Ensembl_gene_identifier	Gene_symbol	Donor_#1				Donor_#2				Donor_#3				Score
			K562/A*0201		PBMC		K562/A*0201		PBMC		K562/A*0201		PBMC		
			cognate vs.		cognate vs.		cognate vs.		cognate vs.		cognate vs.		cognate vs.		
			DMSO	mock	DMSO	mock	DMSO	mock	DMSO	mock	DMSO	mock	DMSO	mock	
1	ENSG00000049249	**TNFRSF9**	S	S	S	S	S	S	S	S	S	S	S	S	18
2	ENSG00000162924	**REL**	S	S	S	S	S	S	S	S	S	S	S	S	18
3	ENSG00000122877	**EGR2**	S	S	S	S	S	S	S	S	S	S	S	S	18
4	ENSG00000140319	**SRP14**	S	S	S	S	S	S	S	S	S	S	S	S	18
5	ENSG00000117560	**FASLG**	S	S	S	S	S	S	S	S	S	S	S	S	18
6	ENSG00000100453	**GZMB**	S	S	S	S	S	S	S	S	S	S	S	S	18
7	ENSG00000111537	**IFNG**	S	S	S	S	S	S	S	S	S	S	NS	S	16.5
8	ENSG00000189403	**HMGB1**	S	S	S	S	S	S	S	S	S	S	NS	S	16.5
9	ENSG00000110848	**CD69**	S	S	S	S	NS	S	S	S	S	S	S	S	16.5
10	ENSG00000168685	**IL7R**	S	S	S	S	S	S	S	S	S	NS	S	S	16.5
11	ENSG00000166886	**NAB2**	S	S	S	S	NS	S	S	S	S	S	S	S	16.5
12	ENSG00000143333	**RGS16**	S	S	S	S	S	S	S	S	NS	NS	S	S	15
13	ENSG00000172116	**CD8B**	S	S	S	S	S	S	S	S	S	S	NS	NS	15
14	ENSG00000140968	**IRF8**	S	S	S	S	S	S	S	S	S	S	NS	NS	15
15	ENSG00000168610	**STAT3**	S	S	S	S	S	S	NS	S	S	S	NS	NS	13.5
16	ENSG00000198851	**CD3E**	S	S	S	S	S	S	S	S	NS	S	NS	NS	13.5
17	ENSG00000134460	**IL2RA**	S	S	NS	NS	S	S	S	S	NS	S	S	S	13.5
18	ENSG00000171791	**BCL2**	S	S	S	S	NS	S	NS	NS	S	S	NS	S	12
19	ENSG00000139193	**CD27**	S	S	S	S	S	S	S	S	NS	NS	NS	NS	8
20	ENSG00000271503	**CCL5**	S	S	NS	S	NS	S	NS	NS	NS	S	NS	NS	7.5
21	ENSG00000275302	**CCL4**	S	S	S	S	S	S	S	NS	NS	NS	NS	NS	7
22	ENSG00000180644	**PRF1**	S	S	NS	S	NS	NS	NS	NS	S	S	S	S	7
23	ENSG00000169442	**CD52**	S	S	S	S	S	S	NS	NS	NS	NS	NS	NS	6
24	ENSG00000073861	**TBX21**	S	S	S	S	NS	NS	NS	NS	S	S	NS	NS	6
25	ENSG00000228978	**TNF**	S	NS	S	S	S	S	NS	S	NS	NS	NS	NS	6
26	ENSG00000135048	**CEMIP2**	S	S	NS	NS	S	NS	NS	NS	S	NS	NS	NS	6
27	ENSG00000075426	**FOSL2**	S	S	NS	NS	S	NS	NS	NS	S	NS	NS	NS	6
28	ENSG00000188486	**H2AFX**	S	S	S	NS	NS	NS	S	S	NS	NS	NS	NS	5
29	ENSG00000115607	**IL18RAP**	S	S	S	NS	NS	NS	NS	NS	S	NS	S	NS	5
30	ENSG00000142166	**IFNAR1**	S	S	NS	S	NS	NS	NS	NS	S	S	NS	NS	5
31	ENSG00000163513	**TGFBR2**	S	NS	S	S	NS	NS	S	S	NS	NS	NS	NS	5
32	ENSG00000121858	**TNFSF10**	S	S	S	S	NS	NS	NS	NS	NS	NS	NS	NS	2
33	ENSG00000087586	**AURKA**	S	NS	NS	NS	NS	NS	NS	S	NS	NS	NS	NS	2
34	ENSG00000109471	**IL2**	S	S	NS	NS	NS	NS	NS	NS	NS	NS	NS	NS	1
35	ENSG00000100450	**GZMH**	NS	S	S	NS	NS	NS	NS	NS	NS	NS	NS	NS	1
36	ENSG00000164400	**CSF2**	S	S	NS	NS	NS	NS	NS	NS	NS	NS	NS	NS	1
37	ENSG00000126353	**CCR7**	S	S	NS	NS	NS	NS	NS	NS	NS	NS	NS	NS	1
38	ENSG00000170345	**FOS**	S	S	NS	NS	NS	NS	NS	NS	NS	NS	NS	NS	1
39	ENSG00000124813	**RUNX2**	S	NS	NS	NS	NS	NS	NS	NS	NS	NS	NS	NS	0.5
40	ENSG00000159216	**RUNX1**	S	NS	NS	NS	NS	NS	NS	NS	NS	NS	NS	NS	0.5
41	ENSG00000105329	**TGFB1**	NS	NS	NS	NS	NS	NS	NS	S	NS	NS	NS	NS	0.5
42	ENSG00000198821	**CD247**	NS	NS	NS	NS	NS	NS	NS	S	NS	NS	NS	NS	0.5
43	ENSG00000113916	**BCL6**	NS	NS	NS	NS	NS	NS	NS	NS	NS	NS	NS	S	0.5
44	ENSG00000145649	**GZMA**	NS	NS	NS	NS	NS	NS	NS	S	NS	NS	NS	NS	0.5
45	ENSG00000026103	**FAS**	NS	NS	NS	NS	NS	S	NS	NS	NS	NS	NS	NS	0.5
46	ENSG00000126456	**IRF3**	NS	NS	NS	NS	NS	NS	NS	NS	S	NS	NS	NS	0.5
47	ENSG00000163600	**ICOS**	NS	NS	NS	NS	NS	NS	NS	NS	NS	NS	NS	NS	0
48	ENSG00000163599	**CTLA4**	NS	NS	NS	NS	NS	NS	NS	NS	NS	NS	NS	NS	0
49	ENSG00000160791	**CCR5**	NS	NS	NS	NS	NS	NS	NS	NS	NS	NS	NS	NS	0
50	ENSG00000177606	**JUN**	NS	NS	NS	NS	NS	NS	NS	NS	NS	NS	NS	NS	0
51	ENSG00000106546	**AHR**	NS	NS	NS	NS	NS	NS	NS	NS	NS	NS	NS	NS	0
52	ENSG00000197919	**IFNA1**	NS	NS	NS	NS	NS	NS	NS	NS	NS	NS	NS	NS	0
53	ENSG00000150907	**FOXO1**	NS	NS	NS	NS	NS	NS	NS	NS	NS	NS	NS	NS	0
54	ENSG00000163508	**EOMES**	NS	NS	NS	NS	NS	NS	NS	NS	NS	NS	NS	NS	0
55	ENSG00000183813	**CCR4**	NS	NS	NS	NS	NS	NS	NS	NS	NS	NS	NS	NS	0
56	ENSG00000178573	**MAF**	NS	NS	NS	NS	NS	NS	NS	NS	NS	NS	NS	NS	0
57	ENSG00000069667	**RORA**	NS	NS	NS	NS	NS	NS	NS	NS	NS	NS	NS	NS	0
58	ENSG00000160712	**IL6R**	NS	NS	NS	NS	NS	NS	NS	NS	NS	NS	NS	NS	0
59	ENSG00000102245	**CD40LG**	NS	NS	NS	NS	NS	NS	NS	NS	NS	NS	NS	NS	0
60	ENSG00000030419	**IKZF2**	NS	NS	NS	NS	NS	NS	NS	NS	NS	NS	NS	NS	0
61	ENSG00000136634	**IL10**	NS	NS	NS	NS	NS	NS	NS	NS	NS	NS	NS	NS	0
62	ENSG00000049768	**FOXP3**	NS	NS	NS	NS	NS	NS	NS	NS	NS	NS	NS	NS	0
63	ENSG00000186891	**TNFRSF18**	NS	NS	NS	NS	NS	NS	NS	NS	NS	NS	NS	NS	0
64	ENSG00000127318	**IL22**	NS	NS	NS	NS	NS	NS	NS	NS	NS	NS	NS	NS	0
65	ENSG00000120738	**EGR1**	NS	NS	NS	NS	NS	NS	NS	NS	NS	NS	NS	NS	0
66	ENSG00000175063	**UBE2C**	NS	NS	NS	NS	NS	NS	NS	NS	NS	NS	NS	NS	0
67	ENSG00000112116	**IL17F**	NS	NS	NS	NS	NS	NS	NS	NS	NS	NS	NS	NS	0
68	ENSG00000105639	**JAK3**	NS	NS	NS	NS	NS	NS	NS	NS	NS	NS	NS	NS	0
69	ENSG00000145839	**IL9**	NS	NS	NS	NS	NS	NS	NS	NS	NS	NS	NS	NS	0
70	ENSG00000113520	**IL4**	NS	NS	NS	NS	NS	NS	NS	NS	NS	NS	NS	NS	0
71	ENSG00000112486	**CCR6**	NS	NS	NS	NS	NS	NS	NS	NS	NS	NS	NS	NS	0
72	ENSG00000112115	**IL17A**	NS	NS	NS	NS	NS	NS	NS	NS	NS	NS	NS	NS	0
73	ENSG00000138684	**IL21**	NS	NS	NS	NS	NS	NS	NS	NS	NS	NS	NS	NS	0
74	ENSG00000169194	**IL13**	NS	NS	NS	NS	NS	NS	NS	NS	NS	NS	NS	NS	0
75	ENSG00000010610	**CD4**	NS	NS	NS	NS	NS	NS	NS	NS	NS	NS	NS	NS	0

Table S1: Overview of statistical results of the targeted gene expression analysis. qPCR-based single-cell gene expression data of Flu $MP_{58\text{-}66}$-directed cells from donors #1–3 stimulated with cognate peptide or control stimuli (solvent DMSO or mock peptide) in the dye-based $CD8^+$ T cell activation assay were compared using the Hurdle model (*40*). Genes were ranked using scores that considered the number of comparisons suggesting significantly different gene expression levels and the number of donors with statistically significant comparisons. Genes with the same score were ranked based on the delta for their median expression differences between the cognate and control stimuli. S = significant; NS = not significant.
