# Supplementary material for: Gene Expression-Based Identification of Antigen-Responsive CD8+ T Cells on a Single-Cell Level
Source: Front Immunol. 2019 Nov 6;10:2568. doi: 10.3389/fimmu.2019.02568 (PMC6851025; doi:10.3389/fimmu.2019.02568)
Supplement: Table S9 — Primer pairs used for preamplification and qPCR. [file Table_9.pdf]

Table S9

Transcript_Id	Ensembl_Gene_Id	Gene_symbol	Primer preamplification 5'	Primer preamplification 3'	Primer qPCR 5'	Primer qPCR 3'
ENST00000242057.8	ENSG00000106546	AHR	TAAAGCCAATCCCAGCTGAA	GACGCTGAGCCTAAGAACTGA	TAAAGCCAATCCCAGCTGAA	GACGCTGAGCCTAAGAACTGA
ENST00000398117.1	ENSG00000171791	BCL2	GAGTTCGGTGGGGTCATGT	ACAGCCAGGAGAAATCAAACA	GAGTTCGGTGGGGTCATGT	ACAGCCAGGAGAAATCAAACA
ENST00000406870.6	ENSG00000113916	BCL6	AGCCGTGAGCAGTTTAGAGC	AAGTCCAGGAGGATGCAGAA	AGCCGTGAGCAGTTTAGAGC	AAGTCCAGGAGGATGCAGAA
ENST00000615863.1	ENSG00000275302	CCL4	CTGTCCTGTCTCTCCTCATGC	GCTTGCTTCTTTTGGTTTGG	TAGCTGCCTTCTGCTCTCCA	GCTTGCTTCTTTTGGTTTGG
ENST00000603197.5	ENSG00000271503	CCL5	CGCTGTCATCCTCATTGCTA	ACACACTTGGCGGTTCTTTC	ATCTGCCTCCCCATATTCCT	ACACACTTGGCGGTTCTTTC
ENST00000330953.5	ENSG00000183813	CCR4	CAAATACAAGCGGCTCAGGT	AGCCCACCAAGTACATCCAG	CAAATACAAGCGGCTCAGGT	AGCCCACCAAGTACATCCAG
ENST00000292303.4	ENSG00000160791	CCR5	GGCCATCTCTGACCTGTTTTT	AAACACAGCATGGACGACAG	GTCCCCTTCTGGGCTCACTA	AAACACAGCATGGACGACAG
ENST00000341935.9	ENSG00000112486	CCR6	TCAGCGATGTTTTCGACTCC	CACCAGAATATTCCCCAGGA	TCAGCGATGTTTTCGACTCC	CACCAGAATATTCCCCAGGA
ENST00000246657.2	ENSG00000126353	CCR7	CAATGAAAAGCGTGCTGGT	ATAGGGAGGAACCAGGCTTT	GTGGTGGCTCTCCTTGTCAT	ATAGGGAGGAACCAGGCTTT
ENST00000266557.3	ENSG00000139193	CD27	CACTACTGGGCTCAGGGAAA	GCGAACGAGAAGACCAGAGT	CTCGTGAAGGACTGTGACCA	GCGAACGAGAAGACCAGAGT
ENST00000361763.8	ENSG00000198851	CD3E	GCACTCACTGGAGAGTTCTGG	CCTCATCACCGCCTATGTTT	GCACTCACTGGAGAGTTCTGG	CCTCATCACCGCCTATGTTT
ENST00000011653.8	ENSG00000010610	CD4	ACCGGGGAGTCCCTTTTAG	CATTCAGCTTGGATGGACCT	ACCGGGGAGTCCCTTTTAG	CATTCAGCTTGGATGGACCT
ENST00000374213.2	ENSG00000169442	CD52	GCGCTTCCTCTTCCTCCTAC	CTGAAGCAGAAGAGGTGGATT	GCGCTTCCTCTTCCTCCTAC	CTGAAGCAGAAGAGGTGGATT
ENST00000390655.10	ENSG00000172116	CD8B	GCTGGACTTCGCCTGTGATAT	TTGTCTCCCGATTTGACCAC	GCTGGACTTCGCCTGTGATAT	TTGTCTCCCGATTTGACCAC
ENST00000296871.3	ENSG00000164400	CSF2	CACTGCTGCTGAGATGAATGA	AGGGCAGTGCTGCTTGTAGT	CACTGCTGCTGAGATGAATGA	AGGGCAGTGCTGCTTGTAGT
ENST00000302823.7	ENSG00000163599	CTLA4	TGACAGCCAGGTGACTGAAG	GTTGCCTATGCCCAGGTAGT	TGGGGAATGAGTTGACCTTC	GTTGCCTATGCCCAGGTAGT
ENST00000242480.3	ENSG00000122877	EGR2	TGGAGAGAAGAGGTCGTTGG	GTTGAAGCTGGGGAAGTGAC	TGGAGAGAAGAGGTCGTTGG	GTTGAAGCTGGGGAAGTGAC
ENST00000295743.8	ENSG00000163508	EOMES	CACAAATACCAACCCCGACT	GGGACAATCTGATGGGATGA	CACAAATACCAACCCCGACT	GGGACAATCTGATGGGATGA
ENST00000355740.6	ENSG00000026103	FAS	CAAGGGATTGGAATTGAGGA	TGGAAGAAAAATGGGCTTTG	ATGGCCAATTCTGCCATAAG	TGGAAGAAAAATGGGCTTTG
ENST00000367721.2	ENSG00000117560	FASLG	GGGATGTTTCAGCTCTTCCA	CAGAGGCATGGACCTTGAGT	CAGAAGGAGCTGGCAGAACT	CAGAGGCATGGACCTTGAGT
ENST00000376207.8	ENSG00000049768	FOXP3	GTAGCCATGGAAACAGCACAT	GCGTGTGAACCAGTGGTAGAT	ACATTCCCAGAGTTCCTCCAC	GCGTGTGAACCAGTGGTAGAT
ENST00000274306.6	ENSG00000145649	GZMA	GAACAAAAGGTCCCAGGTCA	TTTTTGCTTTTTCCATCAGC	GAACAAAAGGTCCCAGGTCA	TTTTTGCTTTTTCCATCAGC
ENST00000216341.8	ENSG00000100453	GZMB	GGTGGCTTCCTGATACGAGA	GCTGCAGTAGCATGATGTCG	ACTGTTGGGGAAGCTCCATA	GCTGCAGTAGCATGATGTCG
ENST00000216338.8	ENSG00000100450	GZMH	CAGCCATTCCTCCTCCTGT	GAGCAGCTGTCAGCACAAAG	TCCTCCTGTTGGCCTTTCTT	GAGCAGCTGTCAGCACAAAG
ENST00000316386.10	ENSG00000163600	ICOS	GGACCATTCTCATGCCAACT	TCGTGCACACTGGATGAATA	GGTTACCCATAGGATGTGCAG	TCGTGCACACTGGATGAATA
ENST00000270139.7	ENSG00000142166	IFNAR1	TGAGTCTGTCGGGAATGTGA	TGCGAAATGGTGTAAATGAGTC	TGAGTCTGTCGGGAATGTGA	TGCGAAATGGTGTAAATGAGTC
ENST00000229135.3	ENSG00000111537	IFNG	CTGTTACTGCCAGGACCCAT	TGGATGCTCTGGTCATCTTT	GGTCATTCAGATGTAGCGGA	TGGATGCTCTGGTCATCTTT
ENST00000434687.5	ENSG00000030419	IKZF2	CGAAAGGGAGCACTCCAATA	ATGGCCCCTGATCTCATCT	CGAAAGGGAGCACTCCAATA	ATGGCCCCTGATCTCATCT
ENST00000423557.1	ENSG00000136634	IL10	TGCTGGAGGACTTTAAGGGTTA	GCCTTGCTCTTGTTTTCACAG	TTTAAGGGTTACCTGGGTTGC	GCCTTGCTCTTGTTTTCACAG
ENST00000304506.7	ENSG00000169194	IL13	GGTCAACATCACCCAGAACC	TTTACAAACTGGGCCACCTC	GTACTGTGCAGCCCTGGAAT	TTTACAAACTGGGCCACCTC
ENST00000340057.1	ENSG00000112115	IL17A	TGGGAAGACCTCATTGGTGT	CCGGTTATGGATGTTCAGGT	TGGGAAGACCTCATTGGTGT	CCGGTTATGGATGTTCAGGT
ENST00000336123.4	ENSG00000112116	IL17F	TCCAAAAGCCTGAGAGTTGC	ATGCAGCCCAAGTTCCTACA	GCCTGTGCCAGGAGGTAGTA	ATGCAGCCCAAGTTCCTACA
ENST00000264260.6	ENSG00000115607	IL18RAP	TTGCAGGAGAGCGAATTAAA	GGTGAGAGTCGATTTCTGTGG	TTGCAGGAGAGCGAATTAAA	GGTGAGAGTCGATTTCTGTGG
ENST00000226730.4	ENSG00000109471	IL2	TGGAGCATTTACTGCTGGATT	GCACTTCCTCCAGAGGTTTG	TGGAGCATTTACTGCTGGATT	GCACTTCCTCCAGAGGTTTG
ENST00000264497.7	ENSG00000138684	IL21	TCGCCACATGATTAGAATGC	AAGCAGGAAAAAGCTGACCA	TCGCCACATGATTAGAATGC	AAGCAGGAAAAAGCTGACCA
ENST00000538666.5	ENSG00000127318	IL22	TCCAGCAGCCCTATATCACC	GTTCAGCACCTGCTTCATCA	TCCAGCAGCCCTATATCACC	GTTCAGCACCTGCTTCATCA
ENST00000231449.6	ENSG00000113520	IL4	TGCCTCCAAGAACACAACTG	CTCTGGTTGGCTTCCTTCAC	GGCAGTTCTACAGCCACCAT	CTCTGGTTGGCTTCCTTCAC
ENST00000303115.7	ENSG00000168685	IL7R	CTGAGGCTCCTTTTGACCTG	CTGCAGGAGTGTCAGCTTTG	CTGAGGCTCCTTTTGACCTG	CTGCAGGAGTGTCAGCTTTG

Table S9: Primer pairs used for preamplification and qPCR.
